# Supplementary material for: Transition from positive to negative indirect CO2 effects on the vegetation carbon uptake
Source: Nat Commun. 2024 Feb 19;15:1500. doi: 10.1038/s41467-024-45957-x (PMC10876672; doi:10.1038/s41467-024-45957-x)
Supplement: Supplementary file 1 — Supplementary Information [file 41467_2024_45957_MOESM1_ESM.pdf]

Supplementary Information for

Transition from positive to negative indirect CO_2 effects on the vegetation carbon uptake

Zefeng Chen[1,2,3], Weiguang Wang[1,2,3*], Giovanni Forzieri[4], Alessandro Cescatti[5]

1. *National Key Laboratory of Water Disaster Prevention, Hohai University, Nanjing, China*
2. *Yangtze Institute for Conservation and Development, Hohai University, Nanjing, China*
3. *College of Hydrology and Water Resources, Hohai University, Nanjing, China*
4. *Department of Civil and Environmental Engineering, University of Florence, Florence, Italy*
5. *European Commission, Joint Research Centre, Ispra, Italy*

*Corresponding author: Dr. Weiguang Wang (wangweiguang@hhu.edu.cn)

Contents of this file

Supplementary Text 1 to 7

Supplementary Figures 1 to 30

Supplementary Tables 1 to 3

Supplementary References

Supplementary Text

Text 1. Bayesian model averaging method

Terrestrial carbon flux projections from different Earth system models (ESMs) exhibit large spread, reflecting the challenge of modelling biogeochemical cycles, and the uncertainty in the prediction of ecosystem responses to future elevated atmospheric CO_2 concentration (eCO_2) and climate[1]. To minimize uncertainties in model simulations, simple model averaging (SMA) method was employed in this study to inherently integrate the model ensemble by giving equal weight to each ESM regardless of its performance. To test if the model-derived findings are affected by the model averaging method and the model ensemble, we confronted SMA-based estimates with those obtained applying the Bayesian model averaging (BMA) method to Coupled Model Intercomparison Project Phase 6 (CMIP6) and Phase 5 (CMIP5) simulations.

BMA has been widely used as optimal estimates for both climate and ecosystem models, and usually performs better than SMA[2]. Different from the "one vote per model" method (i.e., SMA), BMA provides an optimal strategy to integrate model simulations based on individual model performance. For each grid-cell, we determined the optimal integration weights for each ESM according to their performance on simulating growing-season gross primary production (GPP) during the period 1982-2014 under the fully-coupled experiment (i.e., "historical" in CMIP5 and CMIP6). Observed GPP (i.e., GPP_{obs}) derived from a recently developed vegetation index (NIRv) was used to evaluate the performance of each ESM in reproducing historical GPP dynamics (details in Methods). The probability density function (PDF) of growing-season GPP (Δ) from N models (N=7 for CMIP6; N=14 for CMIP5+CMIP6) given observations D (i.e., GPP_{obs}) can be represented as:

$$p(\Delta|D) = \sum_{k=1}^{N} p(\Delta|M_k, D)p(M_k|D) \quad (10)$$

where $p(\Delta|M_k, D)$ is the posterior distribution of Δ given model prediction M_k and observations D, $p(M_k|D)$ is the posterior probability of model prediction M_k which reflects how well this particular ensemble member matches the observations D. This term can be viewed as weight (w_k), and the corresponding sum of w_k equals to 1

$(\sum_{k=1}^{N} p(M_k|D) = \sum_{k=1}^{N} w_k = 1)$[3].

The posterior mean and variance of the ensemble prediction can be expressed as follows:

$$E(\Delta|D) = \sum_{k=1}^{N} w_k M_k \quad (11)$$

$$Var(\Delta|D) = \sum_{k=1}^{N} w_k [M_k - E(\Delta|D)]^2 + \sum_{k=1}^{N} w_k \sigma_k^2 \quad (12)$$

where σ_k^2 is the variance of the model prediction M_k with respect to observation D.

To satisfy the Gaussian assumption, both simulated and observed GPP time-series were first transformed by the Box-Cox method prior to the BMA procedure[4]. The BMA weights and variance were subsequently estimated using the Expectation-Maximization (EM) algorithm, which is iterative and converges to a local maximum likelihood. Spatial pattern of ESMs that were assigned the largest BMA weights is shown in Supplementary Fig. 29. The weights as described above were also used to calculate the ensemble mean of growing-season GPP for all idealized and future scenario experiments at the grid-cell scale.

Text S2. CMIP5 model simulations

To further corroborate CMIP6-based findings, we additionally used outputs from seven ESMs archived as part of carbon-climate feedback experiment within CMIP5[5] (https://esgf-node.llnl.gov/search/cmip5/). The seven ESMs are BCC-CSM1-1, CanESM2, GFDL-ESM2M, GISS-E2-H-CC, GISS-E2-R-CC, HadGEM2-ES, and IPSL-CM5A-LR (Supplementary Table 1). Similar to CMIP6, we analyzed one biogeochemically-coupled experiment and one fully-coupled experiment ("historical" in the CMIP5 terminology) for each CMIP5 model. This biogeochemically-coupled experiment ("esmFixClim2" in the CMIP5 terminology) is forced with changing conditions expect for the radiation code being prescribed with preindustrial CO_2, consistent with the "hist-bgc" experiment within CMIP6. However, "esmFixClim2" experiment runs under RCP4.5 scenario for the future period and therefore different from "ssp585-bgc" experiment that runs under SSP5-8.5 future scenario. For this reason, the seven ESMs from CMIP5 were only applied for the historical period analysis (1982-2014). Although the regular CMIP5 historical experiment ends in 2005,

we extended the CMIP5 model simulations to 2014 by combining the historical experiments (1982-2005) with outputs from the RCP4.5 future scenario from 2006 to 2014, in order to match the historical simulation period in CMIP6. This extending approach has been adopted by numerous studies[6-8]. All CMIP5 simulations were resampled to the common 0.5°×0.5° global grid-cell using the bilinear method of interpolation, and their growing season was identified based on the same definition applied for CMIP6 data (i.e., monthly mean air temperature (T) > 0°C with cumulative precipitation (P) between 10% and 90% of the annual total P, P threshold is only applied to arid and semi-arid regions) (details in Methods).

Text S3. Indirect effect of eCO_2 retrieved from kNDVI

In addition to the NIRv-derived GPP dataset (i.e., GPP_{obs}), we used another satellite GPP proxy, that is the kernel normalized difference vegetation index (kNDVI), to estimate the observed indirect effect of eCO_2 on vegetation carbon uptake and its change during the historical period. Obtained results are then compared with model-based and GPP_{obs}-based estimates to further verify the robustness of our findings. NDVI used in this study was acquired from the third generation dataset of Global Inventory Modeling and Mapping Studies (GIMMS3g v1), which was generated based on the Advanced Very High Resolution Radiometer (AVHRR) reflectance observations[9] (https://ecocast.arc.nasa.gov/data/pub/gimms/3g.v1/). GIMMS3g NDVI was selected since it provides the continuous series with the longest time coverage (start from 1981) among various global NDVI products and successfully covers our entire reference period (1982-2014). Recently, kNDVI has been proposed as a better proxy for ecosystem productivity than the original NDVI, based on flux tower measures of GPP and satellite retrievals of sun-induced fluorescence (SIF)[10]. kNDVI presents a series of advantages compared to standard spectral-based vegetation indices (e.g., NDVI, EVI) including an enhanced resistance to saturation, bias, and complex phenological cycles, as well as a higher robustness to noise and stability across spatial and temporal scales[10]. Recent works have exploited kNDVI and further demonstrated its potential in capturing vegetation dynamics[11]. Following these studies, we have applied kNDVI as a proxy of

GPP (Supplementary Fig. 20b).

kNDVI, as a nonlinear generalization of the NDVI based on Camps-Valls et al.[10], can be derived as follows:

$$\text{kNDVI} = \tanh(\text{NDVI}^2) \tag{13}$$

To be consistent with GPP_{obs} and simulated GPP by seven CMIP6 models, we first composited the original kNDVI (15-day temporal resolution with 1/12° spatial resolution) to the growing-season temporal resolution by averaging the composites within the growing season (Supplementary Fig. 20), and then resampled to the common 0.5° spatial resolution using the bilinear interpolation method. Within the temporal climate analog framework, we calculated the indirect effect of eCO_2 on global vegetation carbon uptake (i.e., $eCO_2(ind)_{kNDVI}$) and its changes between the period 1982-1996 and 2000-2014 based on growing-season-averaged kNDVI.

Results based on kNDVI suggest that global indirect effect of eCO_2 decreases from 0.12 10^{-3} ppm^{-1} during 1982-1996 to -0.11 10^{-3} ppm^{-1} during 2000-2014, whose decreasing magnitude is up to 0.23 10^{-3} ppm^{-1} (Supplementary Fig. 3). Such global weakening effect of eCO_2-driven climate change on vegetation carbon uptake revealed here is consistent with results based on GPP_{obs} and CMIP6 model simulations (Fig. 1). Moreover, $eCO_2(ind)_{kNDVI}$ shows a remarkable decrease in the Northern Hemisphere, prominently over high-latitudes regions (>50°N). An emerging decline can be observed in cold-dry regions in combination with an opposite pattern in warm-wet regions, fully consistent with $eCO_2(ind)_{obs}$ and $eCO_2(ind)$ during the historical period (Fig. 1 and Supplementary Fig. 3). All together, these signals further confirm the robustness of our findings.

Nevertheless, we noted that results based on kNDVI shows a weakening in indirect effect of eCO_2 also in southern low-latitude regions prominently in Brazil (Supplementary Fig. 3b,c). This discrepancy may arise from the relatively large uncertainty of satellite retrievals (both kNDVI and NIRv) in evergreen broad-leaved forests[10].

Text S4. Results from the idealized 1%yr^{-1} increasing CO_2 experiments

In addition to the historical and future scenario (i.e., Shared Socioeconomic Pathways (SSPs)) simulations mentioned in the main text (see Methods), CMIP6 also provides model simulations under an idealized scenario of 1% increase per year in the atmospheric CO_2 concentration. Six of seven ESMs used in this study (ACCESS-ESM1-5, CanESM5, CNRM-ESM2-1, MIROC-ES2L, MRI-ESM2-0, and UKESM1-0-LL) participated in this idealized 1%yr^{-1} increasing CO_2 experiment, and provided simulations run with fully-, biogeochemically-, and radiatively-coupled modes ("1pctCO2", "1pctCO2-bgc" and "1pctCO2-rad" in the CMIP6 terminology, respectively). In the "1pctCO2" experiment, atmospheric CO_2 concentration was prescribed to increase from 285 ppm to 1140 ppm at a rate of 1% per year over a 140-year period, and both radiative and biogeochemical processes respond to increasing CO_2. In the "1pctCO2-bgc" and "1pctCO2-rad" experiments, CO_2 was set to increase at the same pace, but only activated for biogeochemical processes or radiative processes, respectively, with another processes set to a fixed CO_2 concentration at the pre-industrial level. The availability of these three experiments allows to disentangle the direct and indirect effects of eCO_2 on global carbon uptake from model simulations within the same framework through factorial simulations. Direct CO_2 effect under the 1%yr^{-1} increasing CO_2 experiment can be directly estimated from factorial simulations (Eq. (14)), thus replacing other methods (i.e., multiple non-linear regression, see Methods). However, we argued that the increasing rate of CO_2 in "1pctCO2", "1pctCO2-bgc" and "1pctCO2-rad" experiments is substantially higher over the historical period compared to that recorded with observations (Supplementary Fig. 9, and also Fig. 1 in Jones et al.[12]). Such difference therefore limits the comparability between model results obtained under these idealized experiments and results retrieved from observations. To maximize confidence in our results, we still considered crucial to focus on CMIP6 model outputs generated under CO_2 conditions during the historical period that are consistent with observations. Furthermore, we argued that compared with results based on idealized simulations (i.e., "1pctCO2", "1pctCO2-bgc" and "1pctCO2-rad"), our approach provides a more intuitive explanation of the specific

time when the initial positive effect of eCO_2-induced climate change on global vegetation carbon uptake will turn negative (Fig. 2a), and when the negative indirect CO_2 effect will overcome the positive direct CO_2 effect (Supplementary Fig. 8) under no climate policies. In this respect, our approach allows us to communicate key messages to policymakers and relevant communities, ultimately fostering the development of effective climate adaption and mitigation strategies.

While recognizing the limitations of the 1%yr^{-1} increasing CO_2 experiment, their use for the estimation of direct and indirect CO_2 effects through factorial simulations within a consistent framework makes them a useful benchmark to further test the robustness of our finding. To this aim, we performed additional analyses based on CMIP6 model simulations under the idealized 1%yr^{-1} increasing CO_2 experiments, and compared the associated results against those based on historical and future scenario model simulations, described in Methods. We estimated the indirect effect of eCO_2 on growing-season GPP under the 1%yr^{-1} increasing CO_2 experiments (hereafter $eCO_2(ind)_{1\%}$) by using Eq. (3) in combination with simulated GPP in "1pctCO2" and "1pctCO2-bgc" experiments and the prescribed increasing rate of CO_2. Meanwhile, we estimated the direct CO_2 effect under the 1%yr^{-1} increasing CO_2 experiments (hereafter $eCO_2(dir)_{1\%}$) similarly based on the following equation:

$$eCO_2(\mathrm{dir})_{1\%} = \frac{\delta \mathrm{GPP^{FULL}}_{1\%} - \delta \mathrm{GPP^{RAD}}_{1\%}}{\delta \mathrm{CO_2}(1\%)} \tag{14}$$

where $\delta \mathrm{GPP^{FULL}}_{1\%}$ and $\delta \mathrm{GPP^{RAD}}_{1\%}$ are the trends in growing-season GPP in the fully-coupled experiment (i.e., "1pctCO2") and the radiatively-coupled experiment (i.e., "1pctCO2-rad"), respectively; $\delta \mathrm{CO_2}(1\%)$ represents the trend in atmospheric CO_2 concentration in these idealized experiments (blue line in Supplementary Fig. 9). To explore the dynamics of the indirect and direct effects of eCO_2 on vegetation carbon uptake under the 1%yr^{-1} increasing rate of atmospheric CO_2 concentration, we calculated the changes in $eCO_2(ind)_{1\%}$ and $eCO_2(dir)_{1\%}$ between the Year 14-28 (period from the 14th year to 28th year for the entire 140 years) and the Year 120-134. These two periods were selected because mean annual atmospheric CO_2 concentration during Year 14-28 and Year 120-134 in "1pctCO2" is largely similar to that one during 1982-

1996 and 2086-2100 in historical and future simulations.

An ensemble of six ESMs shows that global $eCO_2(ind)_{1\%}$ decreases significantly by 0.33 gC m^{-2} ppm^{-1} between the Year 14-28 and the Year 120-134 (p<0.01, t-test) (Supplementary Fig. 10a,b). Such decreasing signal is statistically significant (p<0.05) over 48.2% of global vegetated land and prominently in northern high-latitudes, eastern Australia and central Africa (Supplementary Fig. 10c,d). The global mean magnitude and the spatial patterns of simulated changes in $eCO_2(ind)_{1\%}$ between the Year 14-28 and the Year 120-134 are largely consistent with those retrieved from the historical and future simulations (Fig. 2a-c). The discrepancy between these two sets of simulations occur in tropics and particularly in central Africa, suggesting possible divergent variations in tropical carbon-climate feedback under different growth rate of atmospheric CO_2 concentration in these regions. Furthermore, results based on multi-model ensemble mean show that global $eCO_2(dir)_{1\%}$ decreases from 1.54±0.27 gC m^{-2} ppm^{-1} in Year 14-28 to 0.46±0.07 gC m^{-2} ppm^{-1} in Year 120-134 (Supplementary Fig. 11a,b). Spatially, 76.0% of the global vegetated land exhibits a significant (p<0.05) decrease in $eCO_2(dir)_{1\%}$ between the Year 14-28 and the Year 120-134 (Supplementary Fig. 11c). Combining together these concurrent temporal changes (Supplementary Fig. 10c), we found that 69.4% of global vegetated land could experience the same direction of change in $eCO_2(ind)_{1\%}$ and $eCO_2(dir)_{1\%}$ (i.e., "+ +" and "– –" in Supplementary Fig. 11d) between the Year 14-28 and the Year 120-134, while the remaining 30.6% could manifest opposite directions of change (i.e., "+ –" and "– +"). The concurrent decrease in $eCO_2(ind)_{1\%}$ and $eCO_2(dir)_{1\%}$ ("– –") is the most pervasive case and occurs in 53.2% of global vegetated land, which is consistent with results based on historical and future scenario simulations (Fig. 3).

Furthermore, we exploited the "1pctCO2-rad" experiment to further assess the validity of our multiple non-linear regression framework (i.e., Eq. (4)) employed to estimate the direct effect of eCO_2 on vegetation carbon uptake. To this aim, we applied the multiple non-linear regression framework (i.e., Eq. (4)) to simulations obtained run with the fully-coupled mode (i.e., "1pctCO2" experiment), and compared the associated estimates (hereafter $eCO_2(dir)_{1\%\text{-RM}}$) against analogous estimates obtained from the

factorial experiment described above (i.e., $eCO_2(dir)_{1\%}$ calculated based on Eq. (14)). At the global scale, $eCO_2(dir)_{1\%\text{-}RM}$ estimated by multi-model ensemble mean decreases from 1.65±0.31 gC m^{-2} ppm^{-1} in Year 14-28 to 0.53±0.13 gC m^{-2} ppm^{-1} in Year 120-134 (Supplementary Fig. 12a,b). The decreasing magnitude of $eCO_2(dir)_{1\%\text{-}RM}$ (-1.12 gC m^{-2} ppm^{-1}, or -68.0%) is quite similar to that one obtained from $eCO_2(dir)_{1\%}$ (-1.08 gC m^{-2} ppm^{-1}, or -70.0%) (Supplementary Fig. 11a,b), suggesting the high consistency between the two sets of results in terms of global mean level. The global patterns of the direct CO_2 effect in Year 14-28 and in Year 120-134 simulated by the use of the non-linear regression model (i.e., $eCO_2(dir)_{1\%\text{-}RM}$) are also generally consistent with those obtained directly from factorial experiments (i.e., $eCO_2(dir)_{1\%}$), as confirmed by the high significant ($p<0.01$) spatial correlation coefficient (0.78 and 0.64, respectively) computed over the vegetated grid-cells (Supplementary Fig. 12c-f). Moreover, results also show that change in direct CO_2 effect between Year 14-28 and Year 120-134 estimated by the non-linear regression model strongly agrees with that directly derived from factorial experiments, with correlation coefficient reaching 0.72 ($p<0.01$) (Supplementary Figs. 11c and 12g,h).

The high consistency of results obtained from non-linear regression and directly from factorial experiments, as described above, proves the suitability of our methods to investigate the direct effect of eCO_2 on global vegetation carbon uptake. In addition, we compared results based on the climate analog approach (Eq. (7)) against analogous estimates based on multiple non-linear regression (Eq. (4)) and factorial experiments (Eq. (14)) under the 1%yr^{-1} increasing CO_2 experiment (Supplementary Fig. 13). As evident, the three sets of results show good agreement, demonstrating the consistency and comparability of results obtained from three different methods.

Text S5. Estimation of E3SM-1-1 ecological and meteorological variables

Net primary production (NPP) of E3SM-1-1 was calculated as the difference between GPP and autotrophic respiration (R_a). Furthermore, monthly specific humidity (SH) derived from E3SM-1-1 was converted to relative humidity (RH) based on T and air pressure (AP) as follows:

$$RH \approx 0.263SH * AP\left\{\exp\left[\frac{17.67(T-273.16)}{T-29.65}\right]\right\}^{-1} \quad (15)$$

where AP is given in Pa, T is in K, SH and the resulting RH are both dimensionless.

The resulting RH was used as input into Eq. (15) to derive monthly vapor pressure deficit (VPD). To derive monthly T_{max} and T_{min} for the model E3SM-1-1 (the model only provides T), we first built regression functions linking T_{max}-T and T_{min}-T from the other six CMIP6 ESMs for each month at the grid-cell scale. Then, we applied such relationships using T values derived from E3SM-1-1 and retrieved the corresponding T_{max} and T_{min}. We assessed the consistency of the estimated T_{max} and T_{min} with those provided by other six ESMs for the period 1982-2100 and with CRU v4.05 climatic observations for the period 1982-2014, as shown in Supplementary Fig. 30.

Text S6. Calculation of potential evapotranspiration (PET)

The Penman-Monteith algorithm, which is recommended by Food and Agriculture Organization of the United Nations (FAO)[13], was applied in this study to estimate PET. Compared with other widely-used algorithms (e.g., Thornthwaite and Priestley-Taylor), the Penman-Monteith algorithm has an improved representation of the physical processes, by integrating the influence from both radiative and aerodynamic components. It is therefore, typically considered as the most reliable method under a range of climatic conditions[14-16]. Its detailed expression is given below:

$$PET = \frac{0.408\Delta(R_n-G)+P_c\frac{900}{T+273}u_2VPD}{\Delta+P_c(1+0.34u_2)} \quad (16)$$

where Δ is the slope of saturation vapor pressure curve, and can be calculated using T; R_n is the net radiation, quantifiable as the difference between the incoming net shortwave radiation and the ongoing net longwave radiation (i.e., $R_n = R_{ns} - R_{nl}$); G is soil heat flux, negligible for long time step calculation (e.g., daily); Pc is the psychrometric constant, directly derivable from AP; u_2 is the wind speed at 2m, and can be converted from wind speed at other height ($u_2 = u_z\frac{4.87}{\ln(67.8z-5.42)}$, z is the height above ground surface). To fully meet the data requirement of Penman-Monteith algorithm, in addition to the variables mentioned in Methods, we also collected time series of monthly AP, wind speed at 10m (u_{10}), R_{ns}, and R_{nl} as simulated by seven CMIP6 ESMs

under the "historical", "ssp585", "hist-bgc" and "ssp585-bgc" experiments (https://esgf-node.llnl.gov/search/cmip6/), and consistently resampled to the 0.5°×0.5° spatial resolution. Considering that R_{ns} and R_{nl} are not directly provided by E3SM-1-1 and MRI-ESM2-0, we used the incoming shortwave radiation (R_s) and incoming clear-sky shortwave radiation (R_{so}) simulated by these two models combined with the following equations to estimate R_{ns} and R_{nl}:

$$R_{ns} = (1 - \alpha)R_s \tag{17}$$

$$R_{nl} = \sigma \frac{{T_{max}}^4 + {T_{min}}^4}{2}\left(0.34 - 0.14\sqrt{VP}\right)\left(1.35\frac{R_s}{R_{so}} - 0.35\right) \tag{18}$$

where α is albedo, assumed 0.23 for the hypothetical grass reference crop; σ is Stefan-Boltzmann constant (4.903×10^{-9} MJ m^{-2} day^{-1}); VP is the actual water vapor, derivable from T and RH (i.e., $VP = 0.6108e^{\frac{17.27T}{T+237.3}}\frac{RH}{100}$). More details of Penman-Monteith algorithm have been delineated by Allen et al.[13].

We used simulations from seven CMIP6 ESMs as inputs to force Eq. (16), and therefore obtained a 7-member ensemble of monthly PET for each-grid cell during the period 1982-2100. Subsequently, this suite of monthly PET was aggregated to the annual scale, and the resulting annual PET was combined with the simulated P to project the spatiotemporal patterns of aridity index (defined as P/PET) both for the historical and the future periods.

The resulting P/PET was used to identify non-humid regions (including arid and semi-arid classes) (Methods), and was also used as an alternative proxy of terrestrial water availability to explore the intrinsic relationship between changes in eCO_2(ind) and land drying/wetting (Supplementary Fig. 17).

Text S7. Incorporating soil moisture into regression framework

It is believed that soil moisture has a more direct control on vegetation carbon uptake than P[1,17]. We therefore replaced P in the multiple non-linear regression framework described in the main text (i.e., Eq. (4)) with surface soil moisture (SM_{surf}), and correspondingly, developed an additional regression framework:

$$GPP = \beta(CO_2) + C_1(SM_{surf}) + C_2(VPD) + C_3(T_{min} \cdot VPD) + C_4(SM_{surf} \cdot CL) + C_5 + \varepsilon \tag{19}$$

We applied such new multiple non-linear regression (Eq. (19)) to observation-driven datasets, i.e. GPP_{obs}, CRU v4.05 climate dataset, and SM_{surf} provided by the Global Land Evaporation and Amsterdam Model (GLEAM v3.8a)[18], and compared against the original regression (Eq. (4)) with the same inputs, to test whether replacing P with SM_{surf} could improve the ability of the regression model in capturing GPP dynamics.

According to the Akaike Information Criterion (AIC), the corrected Akaike Information Criterion (AICc) and the Bayesian Information Criterion (BIC), we found that Eq. (19) does not lead to a substantial improvement in model performance compared to Eq. (4). On the contrary, for a considerable part of global vegetated land, Eq. (19) has a lower performance than Eq. (4) (Supplementary Fig. 26a-c,g). Specifically speaking, global mean AIC, AICc, and BIC of Eq. (19) reach 160.06, 176.06, and 165.01 for the period 1982-1996, slightly lower than those of Eq. (4) (160.60, 176.60, and 165.55). Spatially, test results show that Eq. (19) has substantially lower AIC, AICc, and BIC (relative change $< -5\%$) in only 5.4%, 4.2%, and 5.0% of global vegetated land than Eq. (4) for the period 1982-1996. By contrast, Eq. (19) has substantially higher AIC, AICc, and BIC (relative change $> 5\%$) in 3.6%, 2.9%, and 3.3% of the globe than Eq. (4). The limited and less widespread decrease in AIC, AICc, and BIC of Eq. (19) compared to Eq. (4) suggests that replacing P with SM_{surf} would not result in a substantial model improvement in representing GPP dynamics. We further compared the observed direct effect of eCO_2 on GPP ($eCO_2(dir)_{obs\text{-}RM}$) estimated by Eq. (19) against analogous estimates obtained from Eq. (4), to assess the potential impact of incorporating soil moisture information on regression results (Supplementary Fig. 27a,b). Results show that $eCO_2(dir)_{obs\text{-}RM}$ estimated by Eq. (19) exhibits a great consistency with that one estimated by Eq. (4) for the periods 1982-1996 and 2000-2014, with spatial correlation coefficient (r) reaching 0.92 (p<0.01), and 0.86 (p<0.01), respectively. The high consistency of results from Eq. (19) and Eq. (4) indicates that replacing P with SM_{surf} has a negligible impact on our results, and further demonstrates the robustness of our estimates of observed direct CO_2 effect.

We also applied Eq. (19) to CMIP6 model simulations under “historical” and

"ssp585" experiments and compared associated results against those based on Eq. (4). In line with those based on observations as mentioned above (Supplementary Fig. 26a-c), results based on ensemble mean of seven ESMs also show that AIC, AICc, and BIC of Eq. (19) are substantially lower (relative change < -5%) than those of Eq. (4) in only 7.6%, 6.7%, and 7.2% of global vegetated land for the period 1982-1996 (Supplementary Fig. 26d-f,g). On the contrary, Eq. (19) has substantially higher AIC, AICc, and BIC (relative change > 5%) in 4.6%, 3.9%, and 4.4% of the vegetated land compared with Eq. (4). Similar conclusions can also be derived in model performance assessments for various periods (e.g., 2000-2014, and 2086-2100). For synthesis purposes, only assessment results for the period 1982-1996 were shown here.

Furthermore, correlation analysis between multi-model means of simulated direct effect of eCO_2 on GPP (eCO_2(dir)) based on Eq. (19) and that one based on Eq. (4) shows Pearson's correlation coefficient of 0.86 (p<0.01), 0.85 (p<0.01), and 0.85 (p<0.01) for the periods 1982-1996, 2000-2014, and 2086-2100, respectively (Supplementary Fig. 27c-e). Apparently, replacing P with SM_{surf} in the regression model has limited impact on our estimates of direct CO_2 effect, regardless of whether the regression model is driven by observation-based datasets or by model outputs.

To test the potential impact of replacing P with SM_{surf} on the inter-model spread, we compared the standard error computed over the seven sets of eCO_2(dir) estimates (corresponding to the seven ESMs) based on Eq. (19) against the analogous estimates based on Eq. (4). Results show that global mean standard error of estimates generated with SM_{surf} is always higher than that one generated with P across all the periods examined in this study (Supplementary Table 3). The larger inter-model spread deriving when replacing P with SM_{surf} in the regression model may be attributed to the difference in representations of the processes related to the soil-vegetation continuum, roots profile and water potentials across ESMs[19,20].

In light of the consideration reported above, in order to minimize inter-model spread and maximize model performance, we adopted P as indicator of water availability conditions in Eq. (4) in our study.

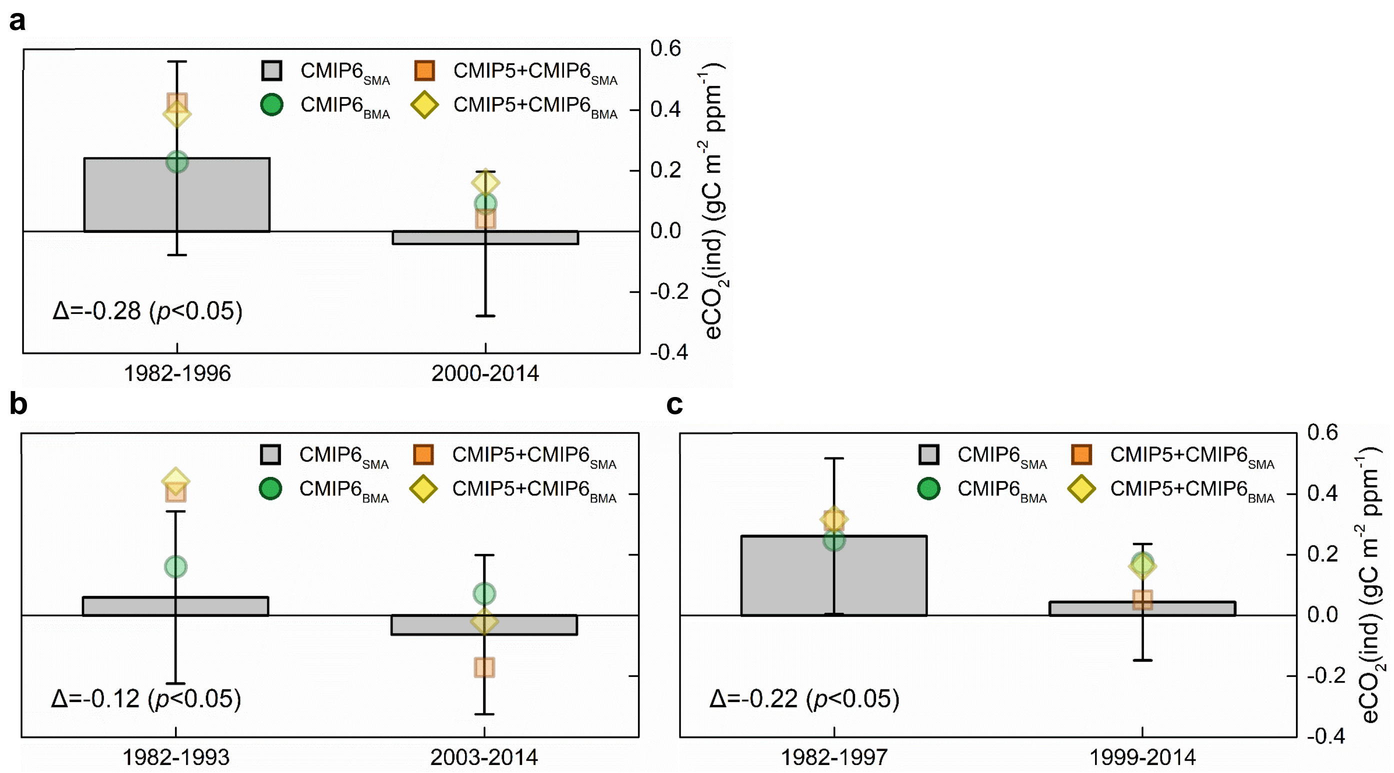

Supplementary Fig. 1 Temporal variations in indirect effect of elevated atmospheric CO_2 concentration (eCO_2) on vegetation carbon uptake derived from different model ensembles and for different temporal window lengths. (a) Mean indirect effect of eCO_2 on growing-season gross primary production (GPP) via associated climate change (eCO_2(ind)) during the periods 1982-1996 and 2000-2014 derived from four different multiple-model ensemble means, respectively. $CMIP6_{SMA}$ represents ensemble of seven CMIP6 ESMs integrated by simple model averaging, and $CMIP5+CMIP6_{BMA}$ represents ensemble of seven CMIP5 ESMs and seven CMIP6 ESMs integrated by Bayesian model averaging, and so on. Error bars represent the standard error of effects derived from members. The number reports the mean of difference in eCO_2(ind) between the two periods. Statistical significance of the difference is assessed by *t*-test. (b) Same as (a), but for eCO_2(ind) during the periods 1982-1993 and 2003-2014. (c) Same as (a), but for eCO_2(ind) during the periods 1982-1997 and 1999-2014. Source data are provided as a Source Data file.

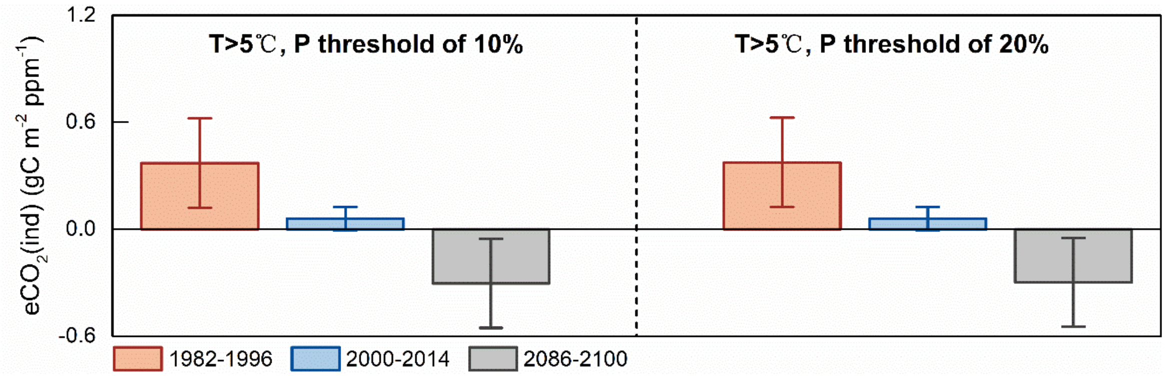

Supplementary Fig. 2 Temporal variations in indirect effect of elevated atmospheric CO_2 concentration (eCO_2) on vegetation carbon uptake obtained based on different definitions of the growing season period. Mean indirect effect of eCO_2 on growing-season gross primary production (GPP) via associated climate change (eCO_2(ind)) during the periods 1982-1996, 2000-2014, and 2086-2100 under the SSP5-8.5 scenario derived from $CMIP6_{SMA}$, based on two definition criterions of the growing season. "T>5°C, P threshold of 10%" represents that under a given temporal window (e.g., 1982-1996), growing season for each vegetated grid-cell was defined as mean monthly temperature (T) >5°C, and exclusively for arid and semi-arid grid-cells (mean annual ratio of annual precipitation to potential evapotranspiration (P/PET) <1), the start of the growing season was defined as the month when the T is >5°C and cumulative P exceeds 10% of the annual total P, and the end of the growing season was defined as the month when the T is >5°C and cumulative P exceeds 90% of the annual total P. Moreover, for arid and semi-arid grid-cells located in Southern Hemisphere, P accumulation was set to start in July and end in June of the next year. Details about the growing season were provided in Methods. Error bars represent the standard error of effects derived from members (i.e., seven CMIP6 ESMs). Source data are provided as a Source Data file.

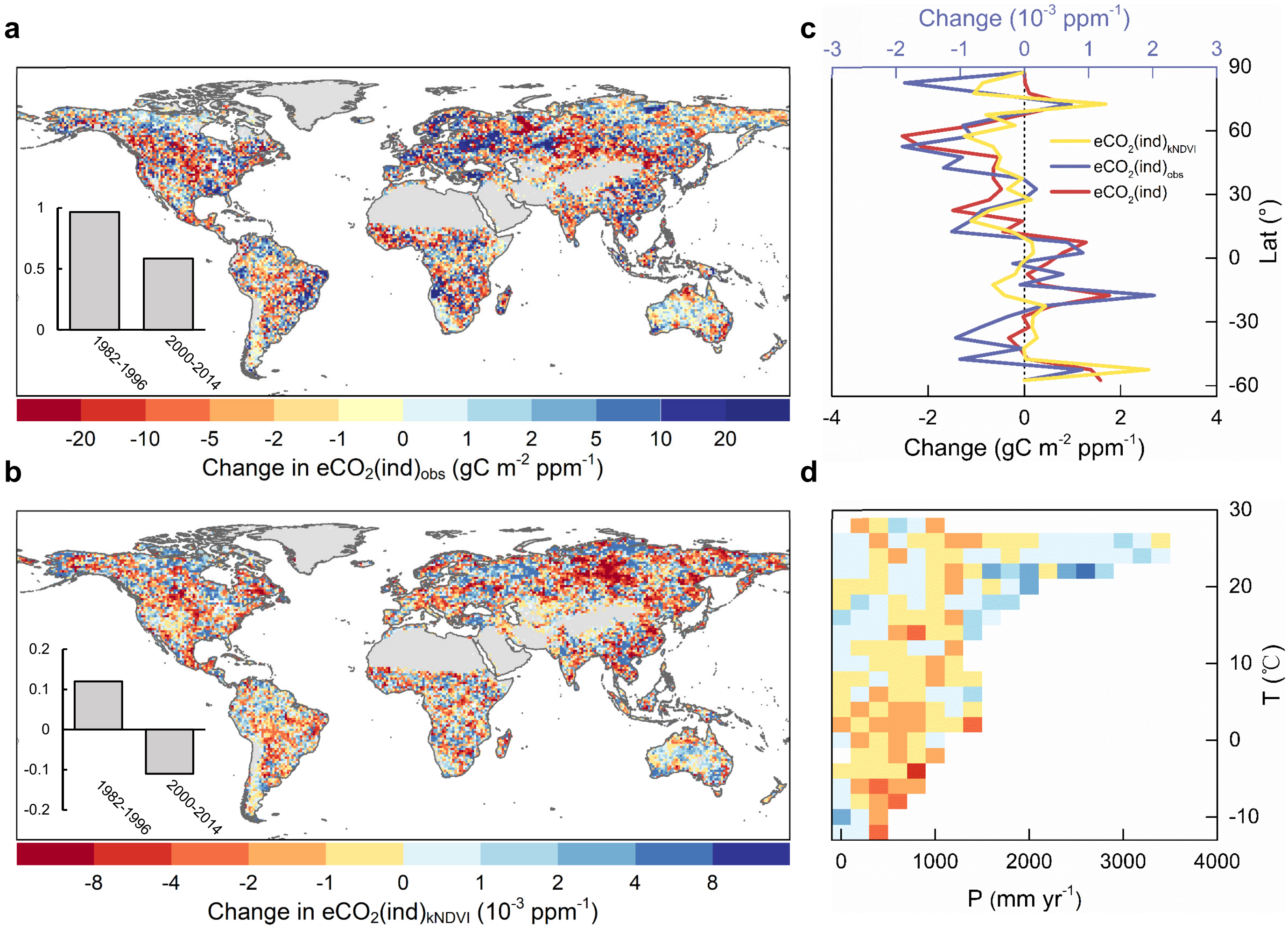

Supplementary Fig. 3 Historical variations in indirect effect of elevated atmospheric CO_2 concentration (eCO_2) on vegetation carbon uptake retrieved from observation-based products. (a) Spatial pattern of difference in observed indirect effect of eCO_2 on growing-season gross primary production (GPP) via associated climate change ($eCO_2(ind)_{obs}$) between the periods 1982-1996 and 2000-2014 based on the growing-season GPP_{obs} under the temporal climate analog framework. Non-vegetated areas or vegetated areas failed to find climate analogous years are excluded in our analysis and are shown in grey (details in Methods). The inset in (a) shows the global mean $eCO_2(ind)_{obs}$ during the periods 1982-1996, and 2000-2014, respectively. (b) Same as (a), but for $eCO_2(ind)_{kNDVI}$ which was based on the growing-season-averaged kernel normalized difference vegetation index (kNDVI). (c) Zonal medians of difference in $eCO_2(ind)_{obs}$, $eCO_2(ind)_{kNDVI}$, and $CMIP6_{SMA}$-simulated $eCO_2(ind)$ between the two periods (2000-2014 versus 1982-1996) at 5° latitudinal resolution. (d) Mean difference in $eCO_2(ind)_{kNDVI}$ between the two periods (2000-2014 versus 1982-1996), binned as a function of climatological mean precipitation (P) and air temperature (T). Source data are provided as a Source Data file.

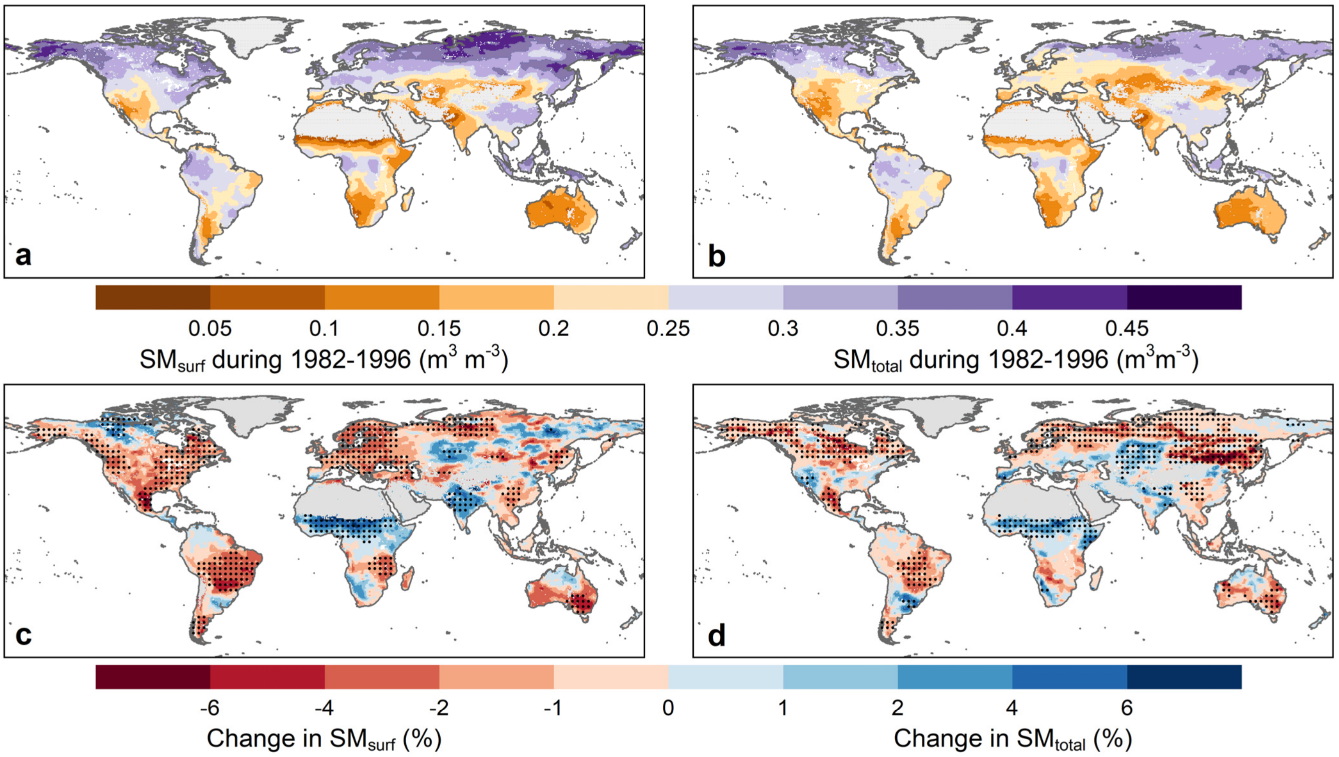

Supplementary Fig. 4 Global soil moisture condition and its variations. (a and b) Spatial pattern of surface soil moisture (SM_{surf}) and total soil moisture (SM_{total}) during the period 1982-1996 derived from $CMIP6_{SMA}$, respectively. (c and d) Spatial pattern of relative changes in SM_{surf} and SM_{total} between the periods 1982-1996 and 2000-2014 derived from $CMIP6_{SMA}$, respectively. Regions labelled by black dots indicate changes that are statistically significant (*t*-test, $p<0.05$). Dots are spaced 3° in both latitude and longitude, and statistics were computed over 9°×9° spatial moving windows.

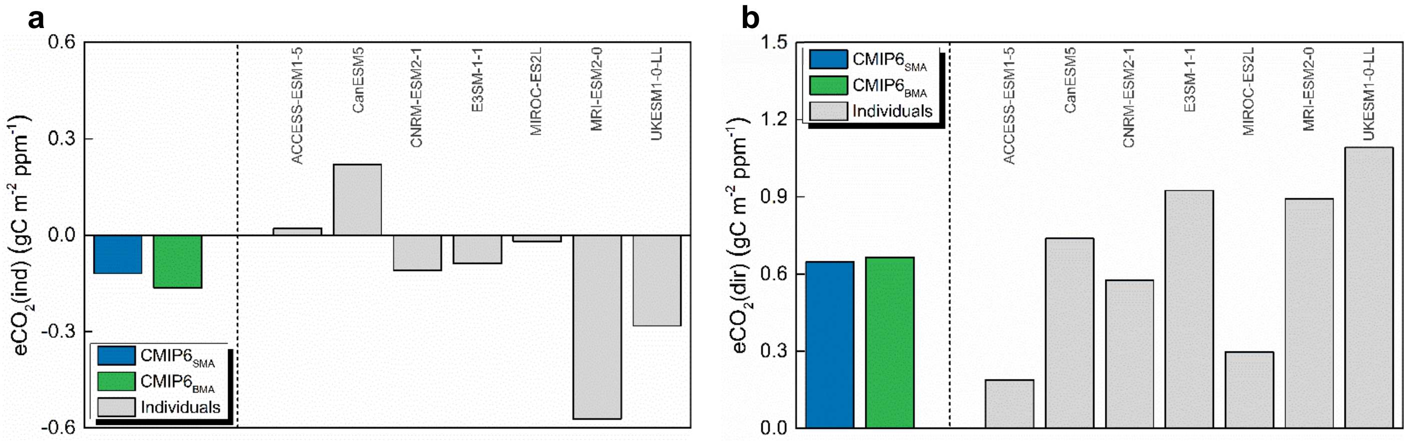

Supplementary Fig. 5 Global mean effects of elevated atmospheric CO_2 concentration (eCO_2) on vegetation carbon uptake during the period 2086-2100 under the SSP5-8.5 scenario. (a) Mean indirect effect of eCO_2 on growing-season gross primary production (GPP) via associated climate change (eCO_2(ind)) for the period 2086-2100 estimated by seven CMIP6 ESMs and their multiple-model ensemble means. (b) Same as (a), but for direct (physiological) effect of eCO_2 on growing-season GPP (eCO_2(dir)). Source data are provided as a Source Data file.

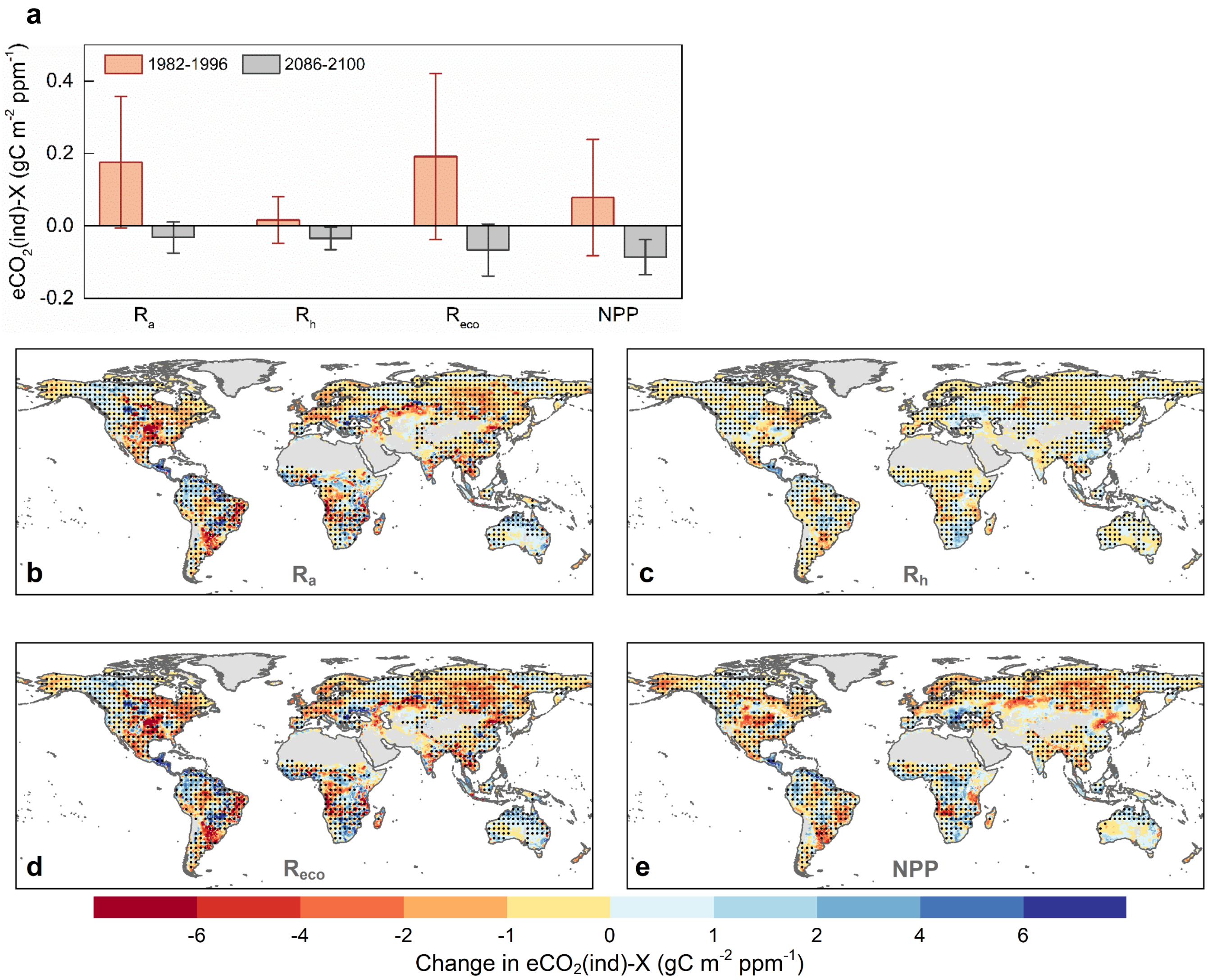

Supplementary Fig. 6 Temporal variations in indirect effect of elevated atmospheric CO_2 concentration (eCO_2) on carbon release and on net ecosystem carbon uptake. (a) Mean indirect effect of eCO_2 on growing-season autotrophic respiration (R_a), heterotrophic respiration (R_h), ecosystem respiration (R_{eco}), and net primary production (NPP) via associated climate change (eCO_2(ind)-X) during the periods 1982-1986 and 2086-2100 under the SSP5-8.5 scenario, derived from $CMIP6_{SMA}$. Error bars represent the standard error of effects derived from members (i.e., seven CMIP6 ESMs). (b) Spatial pattern of difference in eCO_2(ind)-R_a between the historical and future periods (2086-2100 versus 1982-1996) derived from $CMIP6_{SMA}$. Regions labelled by black dots indicate differences that are statistically significant (*t*-test, $p<0.05$). Dots are spaced 3° in both latitude and longitude, and statistics were computed over 9°×9° spatial moving windows. (c-e) Same as (b), but for eCO_2(ind)-R_h, eCO_2(ind)-R_{eco}, eCO_2(ind)-NPP, respectively. Source data are provided as a Source Data file.

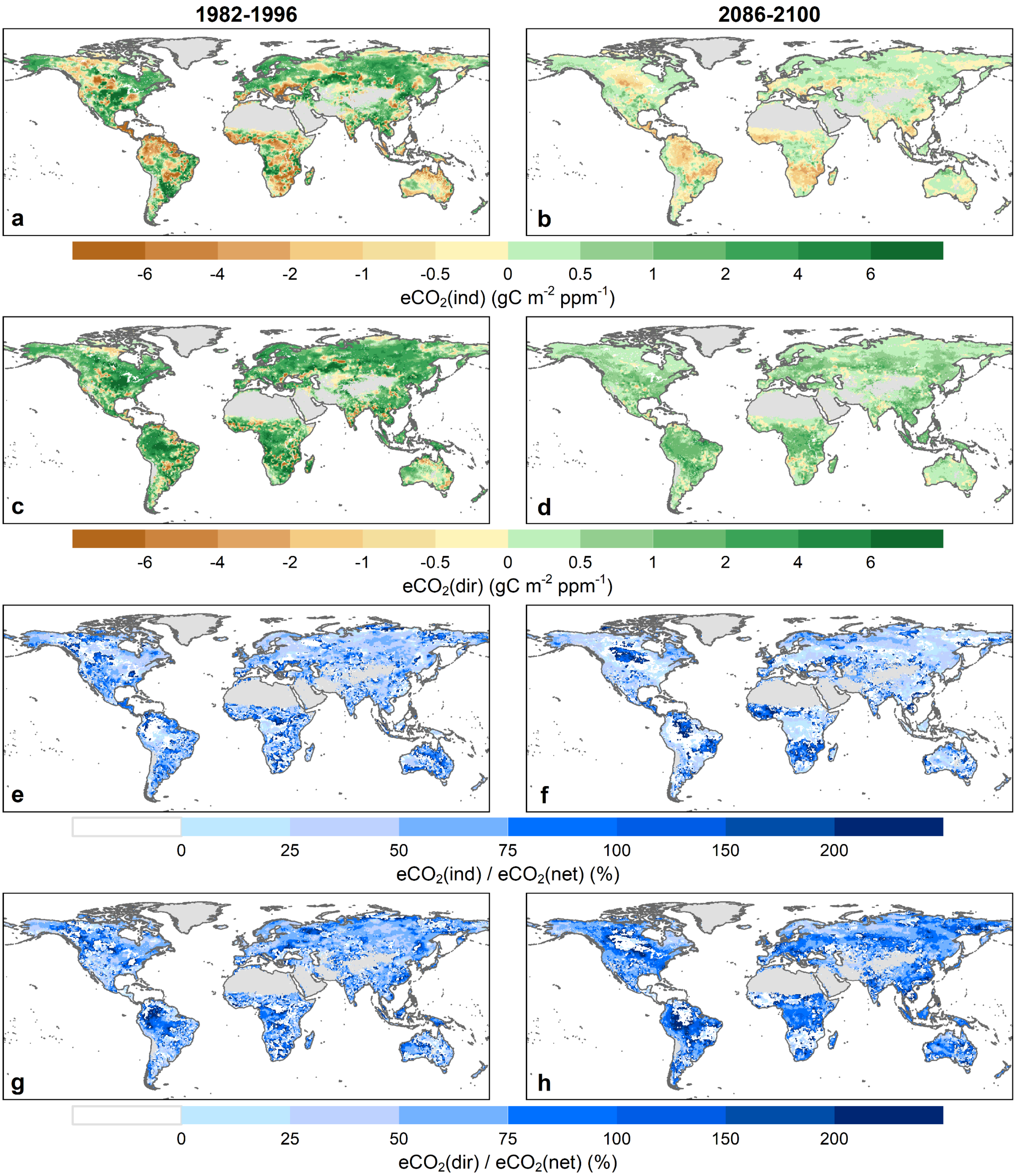

Supplementary Fig. 7 Spatial patterns of effects of elevated atmospheric CO_2 concentration (eCO_2) on vegetation carbon uptake. (a and b) Spatial pattern of indirect effect of eCO_2 on growing-season gross primary production (GPP) via associated climate change (eCO_2(ind)) during the periods 1982-1996 and 2086-2100 under the SSP5-8.5 scenario, derived from $CMIP6_{SMA}$. (c and d) Same as (a and b), but for direct (physiological) effect of eCO_2 on growing-season GPP (eCO_2(dir)). (e and f) Spatial pattern of relative contribution of eCO_2(ind) to net effect of eCO_2 (eCO_2(net)) during the periods 1982-1996 and 2086-2100 under the SSP5-8.5 scenario, derived from $CMIP6_{SMA}$. (g and h) Same as (e and f), but for the relative contribution of eCO_2(dir).

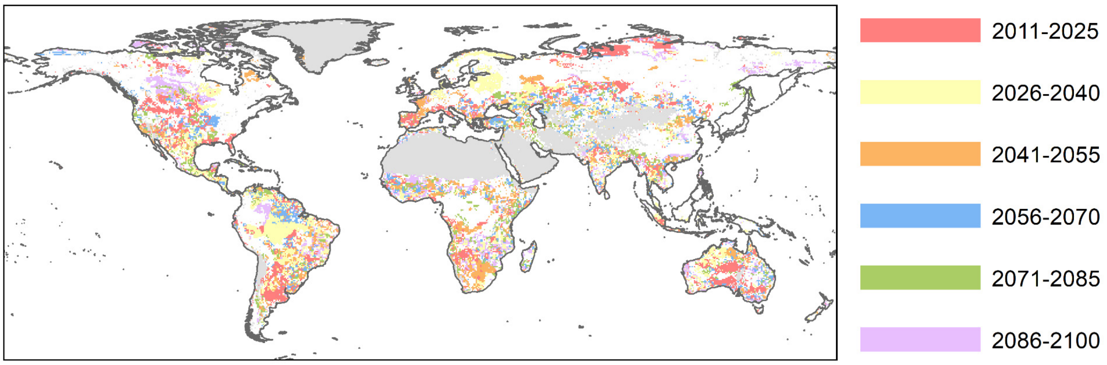

Supplementary Fig. 8 Spatial pattern of the earliest period when negative indirect effect of elevated atmospheric CO_2 concentration (eCO_2) overcomes its positive direct effect on vegetation carbon uptake. Intuitively speaking, such condition can be expressed as $eCO_2(ind)<0$, $eCO_2(dir)>0$, and $|eCO_2(ind)|> |eCO_2(dir)|$, which was projected by $CMIP6_{SMA}$. Vegetated grid-cells without this case during the whole future period (up to 2100) were shown in white.

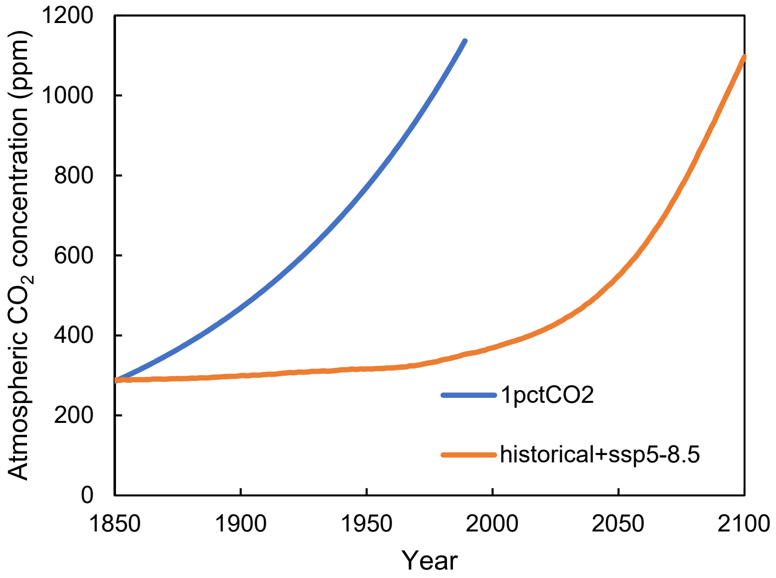

Supplementary Fig. 9 Interannual changes in atmospheric CO_2 concentration at the global scale in idealized 1%yr^{-1} increasing CO_2 simulations (i.e., "1pctCO2") and in historical and future scenario (SSP5-8.5) simulations, respectively. Source data are provided as a Source Data file.

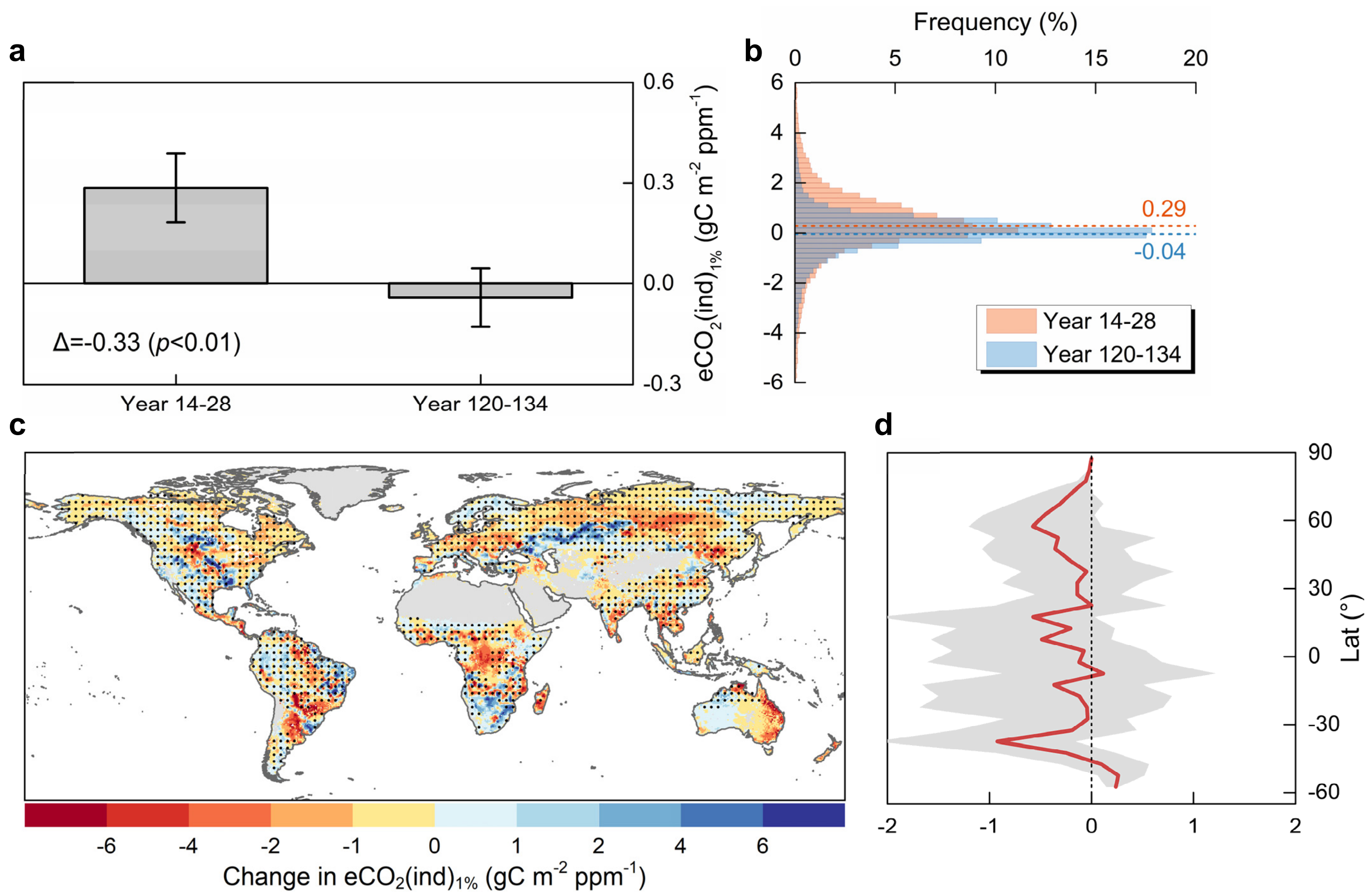

Supplementary Fig. 10 Temporal variations in indirect effect of elevated atmospheric CO_2 concentration (eCO_2) on vegetation carbon uptake in idealized 1%yr^{-1} increasing CO_2 experiments. (a) Mean indirect effect of eCO_2 on growing-season gross primary production (GPP) via associated climate change ($eCO_2(ind)_{1\%}$) during Year 14-28 and Year 120-134, as derived from an ensemble of six ESMs (details in Supplementary Text 4). Error bars represent the standard error of effects derived from ensemble members. Δ expresses the mean of difference in $eCO_2(ind)_{1\%}$ between the two periods. Statistical significance of the difference is assessed by *t*-test. (b) Frequency distribution of $eCO_2(ind)_{1\%}$ at the global scale during Year 14-28 and Year 120-134, as estimated by an ensemble of six ESMs. Distribution averages are shown as dotted horizontal lines. (c) Spatial pattern of difference in $eCO_2(ind)_{1\%}$ between the two periods (Year 120-134 versus Year 14-28) derived from an ensemble of six ESMs. Non-vegetated areas are excluded in our analysis and are shown in grey. Regions labelled by black dots indicate differences that are statistically significant (*t*-test, $p<0.05$). Dots are spaced 3° in both latitude and longitude, and statistics were computed over 9°×9° spatial moving windows. (d) Zonal medians of difference in $eCO_2(ind)_{1\%}$ between the two periods (Year 120-134 versus Year 14-28) simulated by an ensemble of six ESMs at 5° latitudinal resolution. Corresponding interquartile ranges of model simulation are shown as shaded band. Source data are provided as a Source Data file.

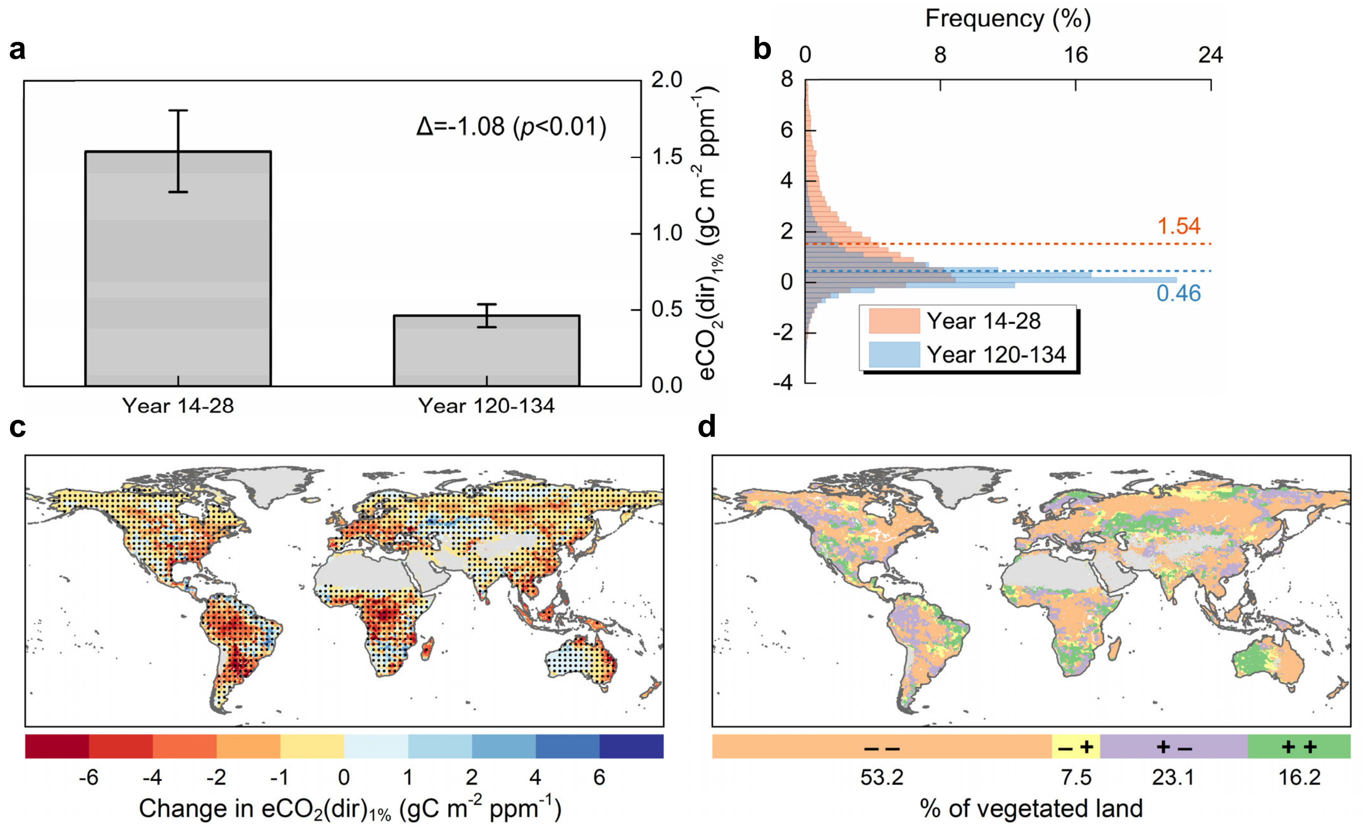

Supplementary Fig. 11 Temporal variations in direct effect of elevated atmospheric CO_2 concentration (eCO_2) on vegetation carbon uptake in idealized 1%yr^{-1} increasing CO_2 experiments. (a) Mean direct (physiological) effect of eCO_2 on growing-season gross primary production (GPP) ($eCO_2(dir)_{1\%}$) during Year 14-28 and Year 120-134, as derived from an ensemble of six ESMs (details in Supplementary Text 4). Error bars represent the standard error of effects derived from ensemble members. Δ expresses the mean of difference in $eCO_2(dir)_{1\%}$ between the two periods. Statistical significance of the difference is assessed by *t*-test. (b) Frequency distribution of $eCO_2(dir)_{1\%}$ at the global scale during Year 14-28 and Year 120-134, as estimated by an ensemble of six ESMs. Distribution averages are shown as dotted horizontal lines. (c) Spatial pattern of difference in $eCO_2(dir)_{1\%}$ between the two periods (Year 120-134 versus Year 14-28) derived from an ensemble of six ESMs. Non-vegetated areas are excluded in our analysis and are shown in grey. Regions labelled by black dots indicate differences that are statistically significant (*t*-test, $p<0.05$). Dots are spaced 3° in both latitude and longitude, and statistics were computed over 9°×9° spatial moving windows. (d) Spatial pattern of relationship between changes in indirect effect of eCO_2 on growing-season GPP via associated climate change ($eCO_2(ind)_{1\%}$) and $eCO_2(dir)_{1\%}$ between the two periods (Year 120-134 versus Year 14-28), where "– –" represents decrease in $eCO_2(ind)_{1\%}$ and decrease in $eCO_2(dir)_{1\%}$, "– +" represents decrease in $eCO_2(ind)_{1\%}$ and increase in $eCO_2(dir)_{1\%}$ and so on. Legend shows the fraction of vegetated areas for each thematic class (i.e., "– –", "– +", "+ –" and "+ +"). Source data are provided as a Source Data file.

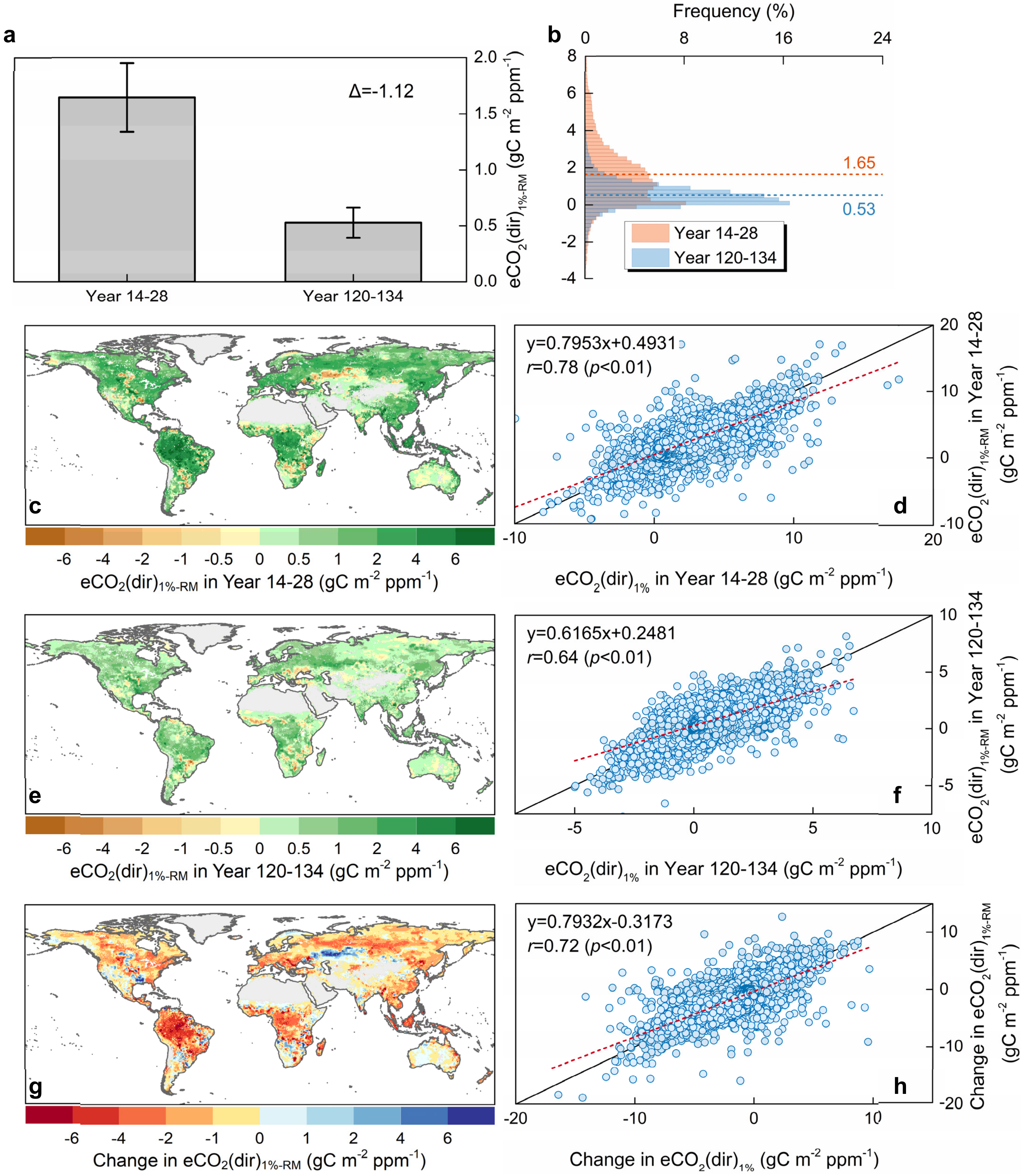

Supplementary Fig. 12 Temporal variations in direct effect of elevated atmospheric CO_2 concentration (eCO_2) on vegetation carbon uptake in idealized 1%yr^{-1} increasing CO_2 experiments estimated by non-linear regression model. (a) Mean direct (physiological) effect of eCO_2 on growing-season gross primary production (GPP) ($eCO_2(dir)_{1\%\text{-RM}}$) during Year 14-28 and Year 120-134, as estimated by multi-model ensemble simulations in combination with the non-linear regression model (i.e., Eq. (4)) (details in Supplementary Text 4). Error bars represent the standard error of effects derived from ensemble members. Δ expresses the mean of difference in $eCO_2(dir)_{1\%\text{-RM}}$ between the two periods. (b) Frequency distribution of $eCO_2(dir)_{1\%\text{-RM}}$ at the global scale during Year 14-28 and Year 120-134. Distribution averages are shown as dotted horizontal lines. (c) Spatial pattern of $eCO_2(dir)_{1\%\text{-RM}}$ during Year 14-28 derived from multi-model ensemble

simulations in combination with the non-linear regression model. Non-vegetated areas are excluded in our analysis and are shown in grey. (d) Comparison of $eCO_2(dir)_{1\%\text{-RM}}$ during Year 14-28 against analogous estimates directly by factorial experiments (i.e., Eq. (14)). Each symbol represents one vegetated grid-cell. Red dotted lines indicate the best-fit with equation provided on each subplot. (e-h) Sames as (c and d), but for $eCO_2(dir)_{1\%\text{-RM}}$ during Year 120-134 and for the difference between the two periods (Year 120-134 versus Year 14-28), respectively. Source data are provided as a Source Data file.

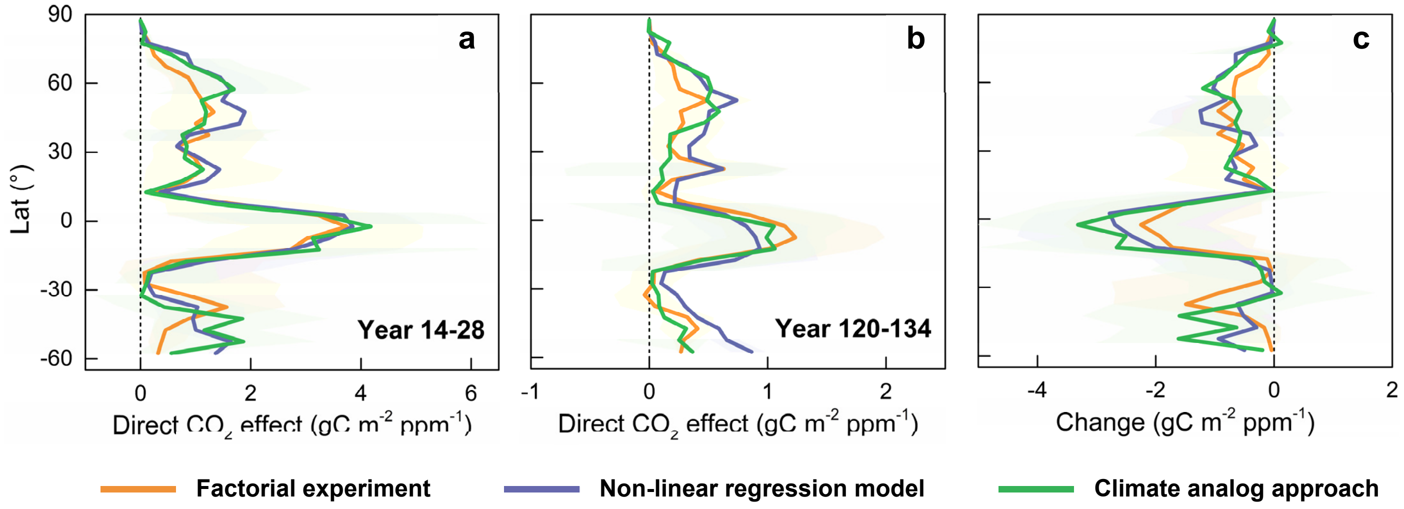

Supplementary Fig. 13 Latitudinal gradients of direct effect of elevated atmospheric CO_2 concentration (eCO_2) on vegetation carbon uptake based on different methods. Zonal medians of direct (physiological) effect of eCO_2 on growing-season gross primary production (GPP) during (a) Year 14-28 and (b) Year 120-134 and (c) their difference (Year 120-134 versus Year 14-28) at 5° latitudinal resolution, as estimated by factorial experiment (Eq. (14)), by non-linear regression model (Eq. (4)) and by climate analog approach (Eq. (7)), respectively. Corresponding interquartile ranges of multi-model simulation are shown as shaded band. Source data are provided as a Source Data file.

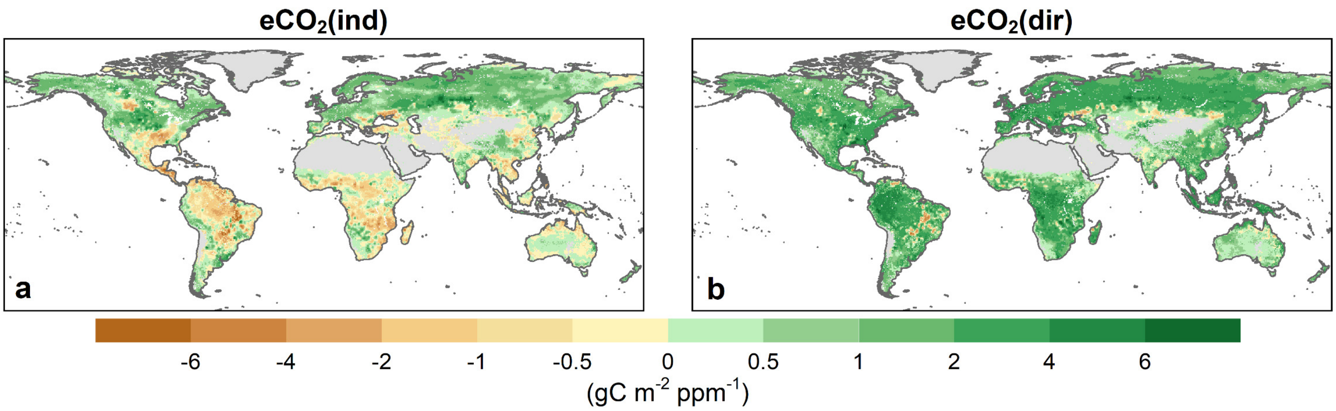

Supplementary Fig. 14 Effects of elevated atmospheric CO_2 concentration (eCO_2) on vegetation carbon uptake during the entire historical period (1982-2014). Spatial pattern of (a) indirect effect of eCO_2 on growing-season gross primary production (GPP) via associated climate change (eCO_2(ind)) and (b) direct (physiological) effect of eCO_2 on growing-season GPP (eCO_2(dir)).

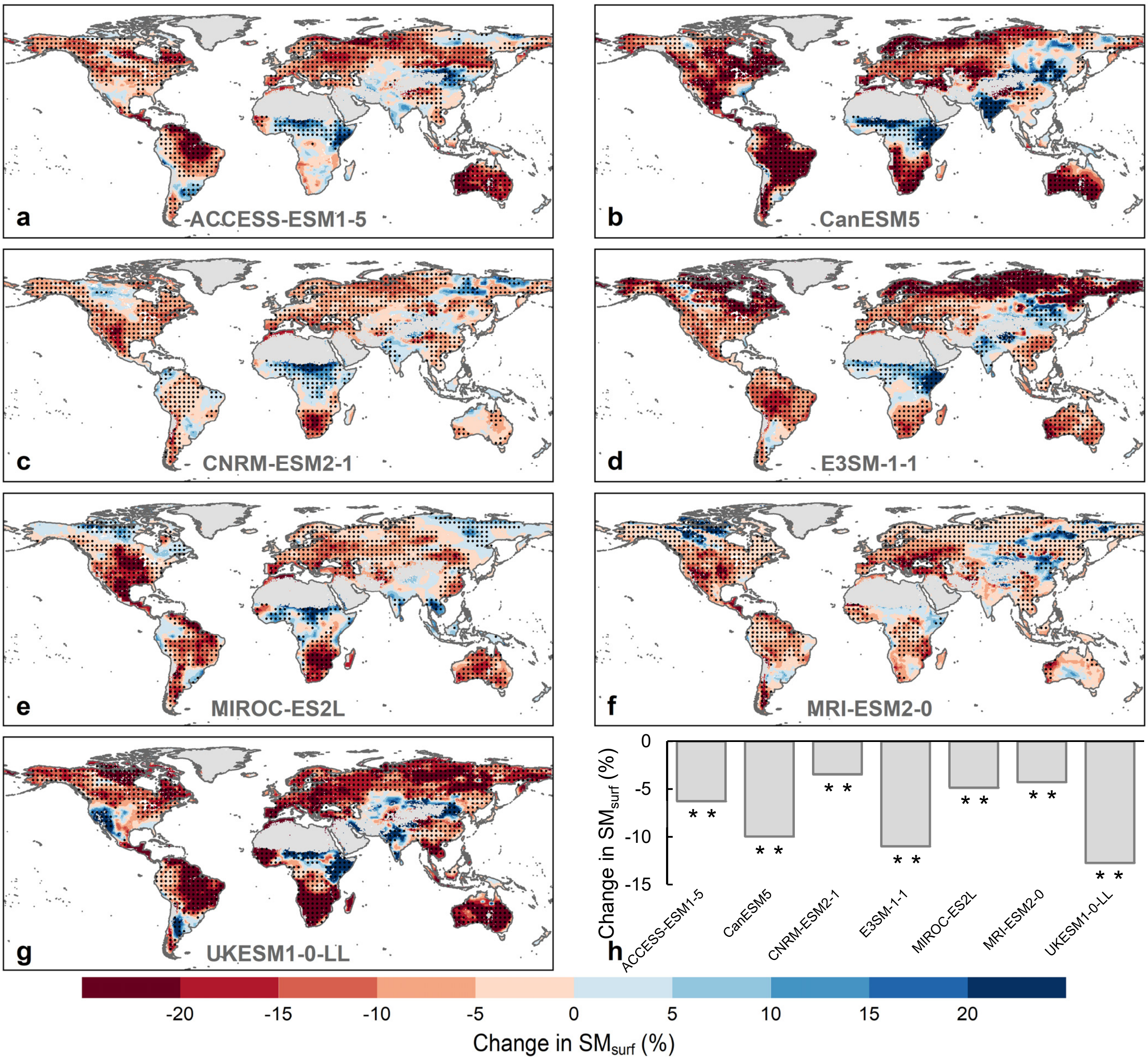

Supplementary Fig. 15 Changes in surface soil moisture (SM_{surf}) at the global scale. (a-g) Spatial pattern of relative change in SM_{surf} between the historical and future periods (2086-2100 versus 1982-1996) derived from seven CMIP6 ESMs, respectively. Regions labelled by black dots indicate changes that are statistically significant (*t*-test, $p<0.05$). Dots are spaced 3° in both latitude and longitude, and statistics were computed over 9°×9° spatial moving windows. (h) Global mean change in SM_{surf} between the historical and future periods derived from seven CMIP6 ESMs, respectively. Two asterisks indicate that the change is statistically significant (*t*-test, $p<0.05$). Source data are provided as a Source Data file.

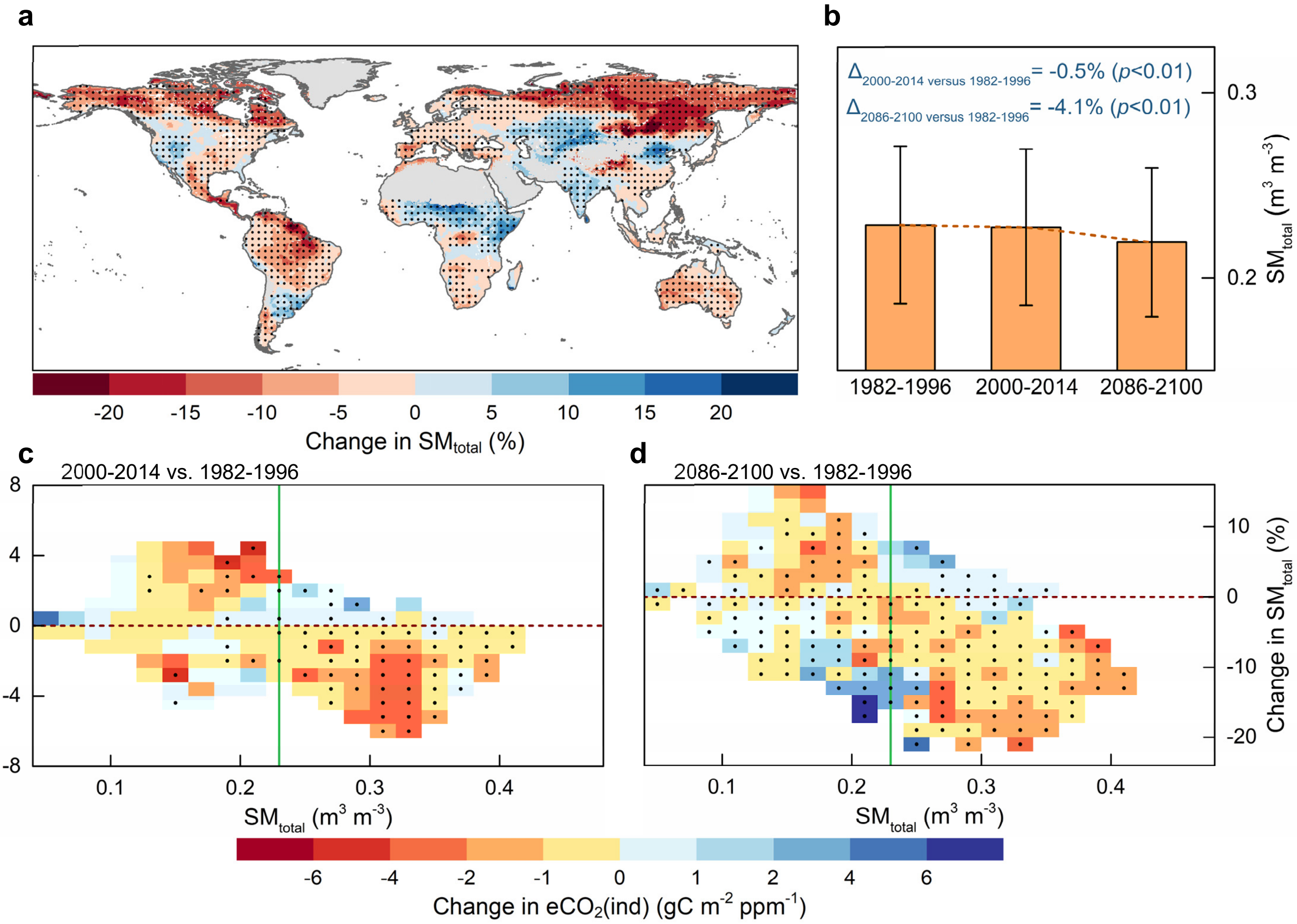

Supplementary Fig. 16 Sensitivity of indirect CO_2 effect on total soil moisture (SM_{total}) condition. (a) Spatial pattern of relative change in SM_{total} between the historical and future periods (2086-2100 versus 1982-1996) derived from $CMIP6_{SMA}$. Regions labelled by black dots indicate changes that are statistically significant (*t*-test, p<0.05). Dots are spaced 3° in both latitude and longitude, and statistics were computed over 9°×9° spatial moving windows. (b) Global mean SM_{total} derived from $CMIP6_{SMA}$ during the period 1982-1996, 2000-2014, and 2086-2100, respectively. Numbers refer to change in SM_{total} relative to 1982-2016. (c) Difference in indirect effect of elevated atmospheric CO_2 concentration (eCO_2) on growing-season gross primary production (GPP) via associated climate change (eCO_2(ind)) between the periods 1982-1996 and 2000-2014 derived from $CMIP6_{SMA}$, binned as a function of corresponding changes in SM_{total} and mean annual SM_{total} (Supplementary Fig. 4b). SM_{total}=0.23 m^3 m^{-3} (i.e., the green solid line) overall corresponds to the ratio of annual precipitation to potential evapotranspiration (P/PET) =1 at the mean annual scale, that is the threshold between non-humid and humid regions (Supplementary Fig. 17b). Black dots indicate bins with differences that are statistically significant (*t*-test, p<0.05). (d) Same as (c), but for difference between the periods 1982-1996 and 2086-2100. Source data are provided as a Source Data file.

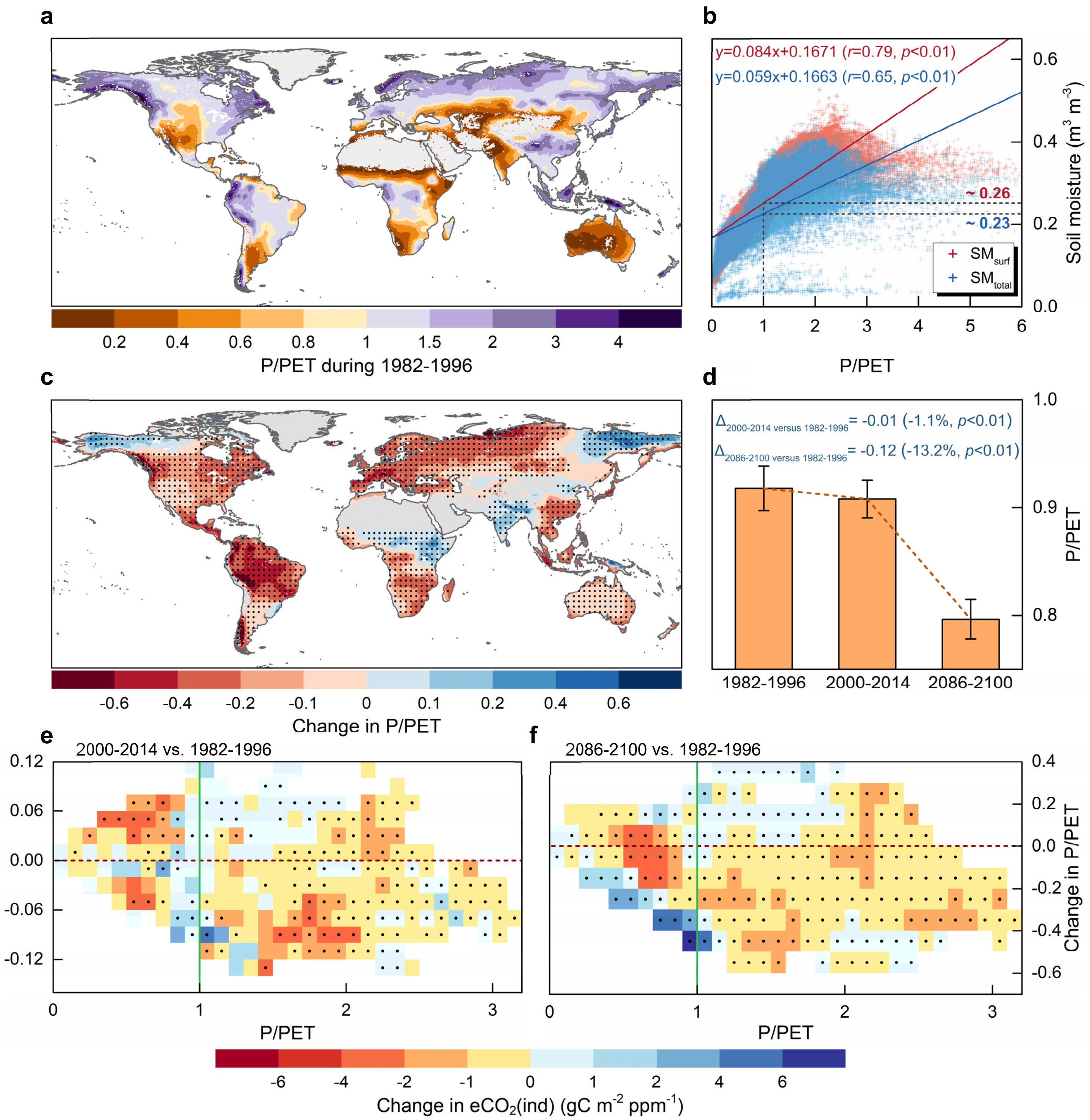

Supplementary Fig. 17 Sensitivity of indirect CO_2 effect on aridity condition (defined as the ratio of mean annual precipitation to potential evapotranspiration, P/PET). (a) Spatial pattern of P/PET during the period 1982-1996 derived from $CMIP6_{SMA}$. (b) Relationship between P/PET and surface soil moisture (SM_{surf}, in red) and total soil moisture (SM_{total}, in blue) during 1982-1996 derived from $CMIP6_{SMA}$. Each symbol represents one vegetated grid-cell. Solid lines indicate the best-fit with equations provided. According to fit equations, P/PET=1, i.e., the threshold between non-humid and humid regions generally corresponds to $SM_{surf} \approx 0.26$ m^3 m^{-3} and $SM_{total} \approx 0.23$ m^3 m^{-3} at the mean annual scale. (c) Spatial pattern of difference in P/PET between the historical and future periods (2086-2100 versus 1982-1996) derived from $CMIP6_{SMA}$. Regions labelled by black dots indicate differences that are statistically significant (*t*-test, $p<0.05$). Dots are spaced 3° in both latitude and longitude, and statistics were computed over 9°×9° spatial moving windows. (d) Global mean P/PET derived from $CMIP6_{SMA}$ during the period 1982-1996, 2000-2014, and 2086-2100, respectively. Numbers refer to change in P/PET relative to 1982-2016. (e) Difference in indirect effect of elevated atmospheric CO_2 concentration (eCO_2) on growing-season

gross primary production (GPP) via associated climate change (eCO_2(ind)) between the periods 1982-1996 and 2000-2014 derived from $CMIP6_{SMA}$, binned as a function of corresponding changes in P/PET and mean annual P/PET in (a). Black dots indicate bins with differences that are statistically significant (*t*-test, $p<0.05$). (f) Same as (e), but for difference between the periods 1982-1996 and 2086-2100. Source data are provided as a Source Data file.

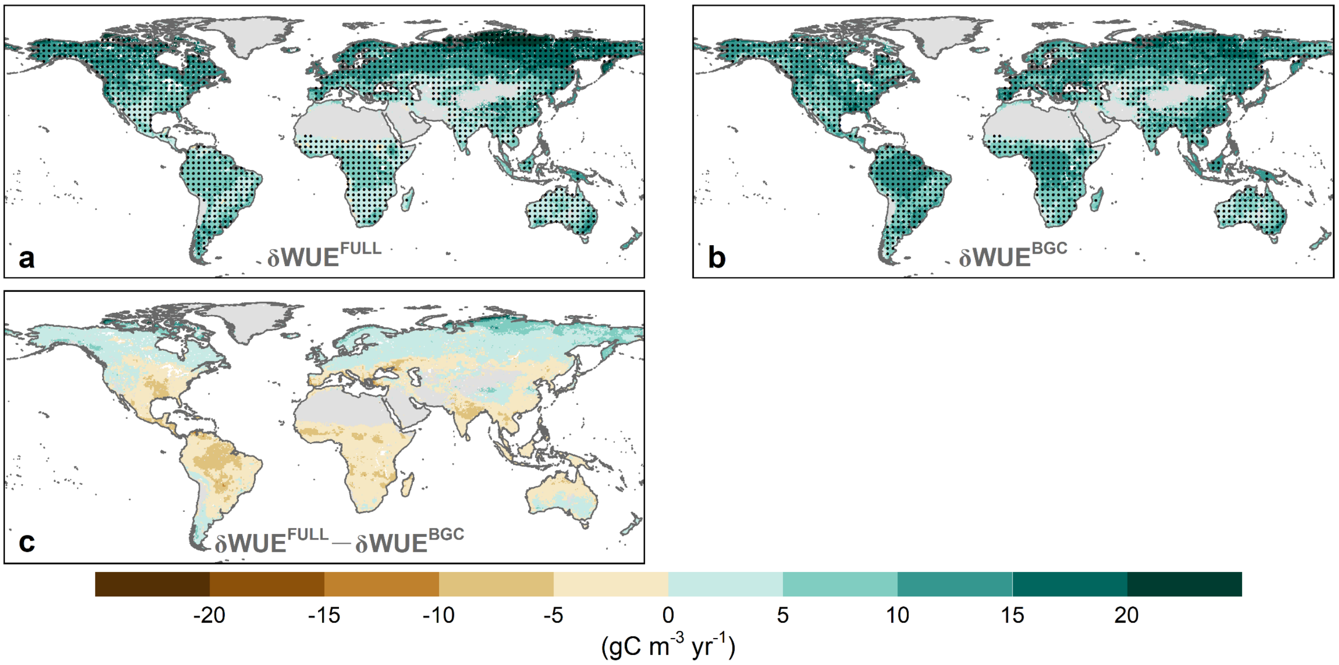

Supplementary Fig. 18 Trend in water-use efficiency (WUE, the ratio of gross primary production to evapotranspiration) for the period 1982-2100. (a and b) Spatial pattern of trend in growing-season WUE during 1982-2100 in the fully-coupled experiment (δWUE^{FULL}) and in the biogeochemically-coupled experiment (δWUE^{BGC}) derived from $CMIP6_{SMA}$. Regions labelled by black dots indicate trends that are statistically significant (Mann–Kendall test, $p<0.05$). Dots are spaced 3° in both latitude and longitude, and statistics were computed over 9°×9° spatial moving windows. (c) Spatial pattern of trend in growing-season WUE in response to CO_2 radiative forcing (δWUE^{FULL}-δWUE^{BGC}) during the period 1982-2100 derived from $CMIP6_{SMA}$.

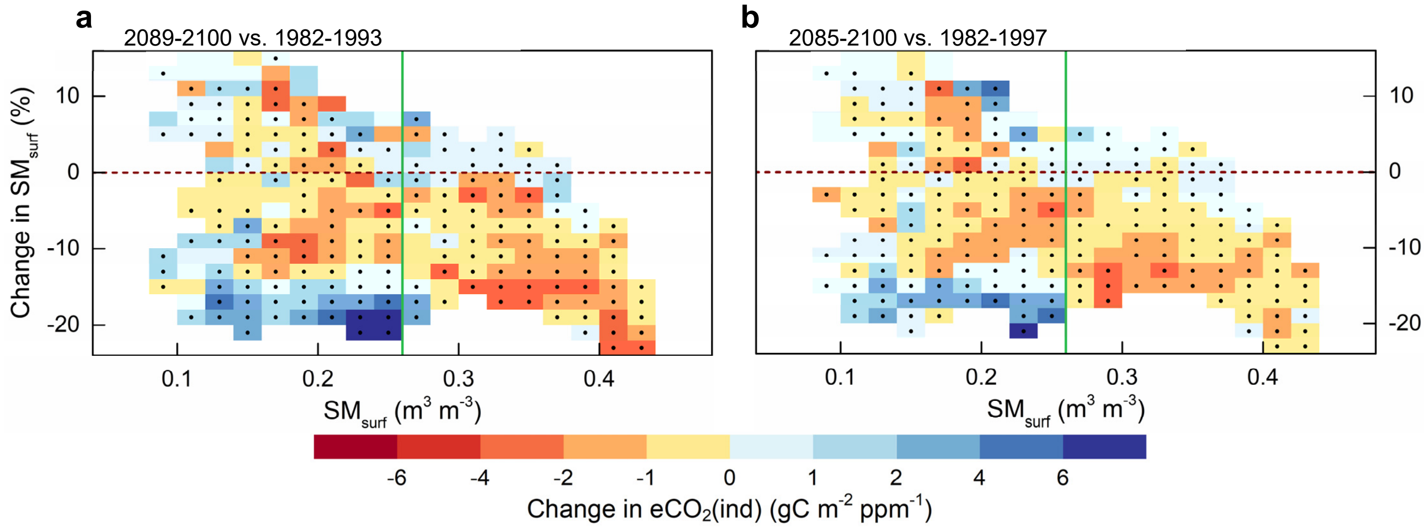

Supplementary Fig. 19 Sensitivity of indirect CO_2 effect on surface soil moisture (SM_{surf}) condition assessed based on different temporal window lengths. (a) Difference in indirect effect of elevated atmospheric CO_2 concentration (eCO_2) on growing-season gross primary production (GPP) via associated climate change (eCO_2(ind)) between the periods 1982-1993 and 2089-2100 derived from $CMIP6_{SMA}$, binned as a function of corresponding changes in SM_{surf} and mean annual SM_{surf} (1982-1993). SM_{surf}=0.26 m^3 m^{-3} (i.e., the green solid line) overall corresponds to the ratio of annual precipitation to potential evapotranspiration (P/PET) =1 at the mean annual scale, that is the threshold between non-humid and humid regions (Supplementary Fig. 17b). Black dots indicate bins with differences that are statistically significant (*t*-test, p<0.05). (b) Same as (a), but for difference between the periods 1982-1997 and 2085-2100. Source data are provided as a Source Data file.

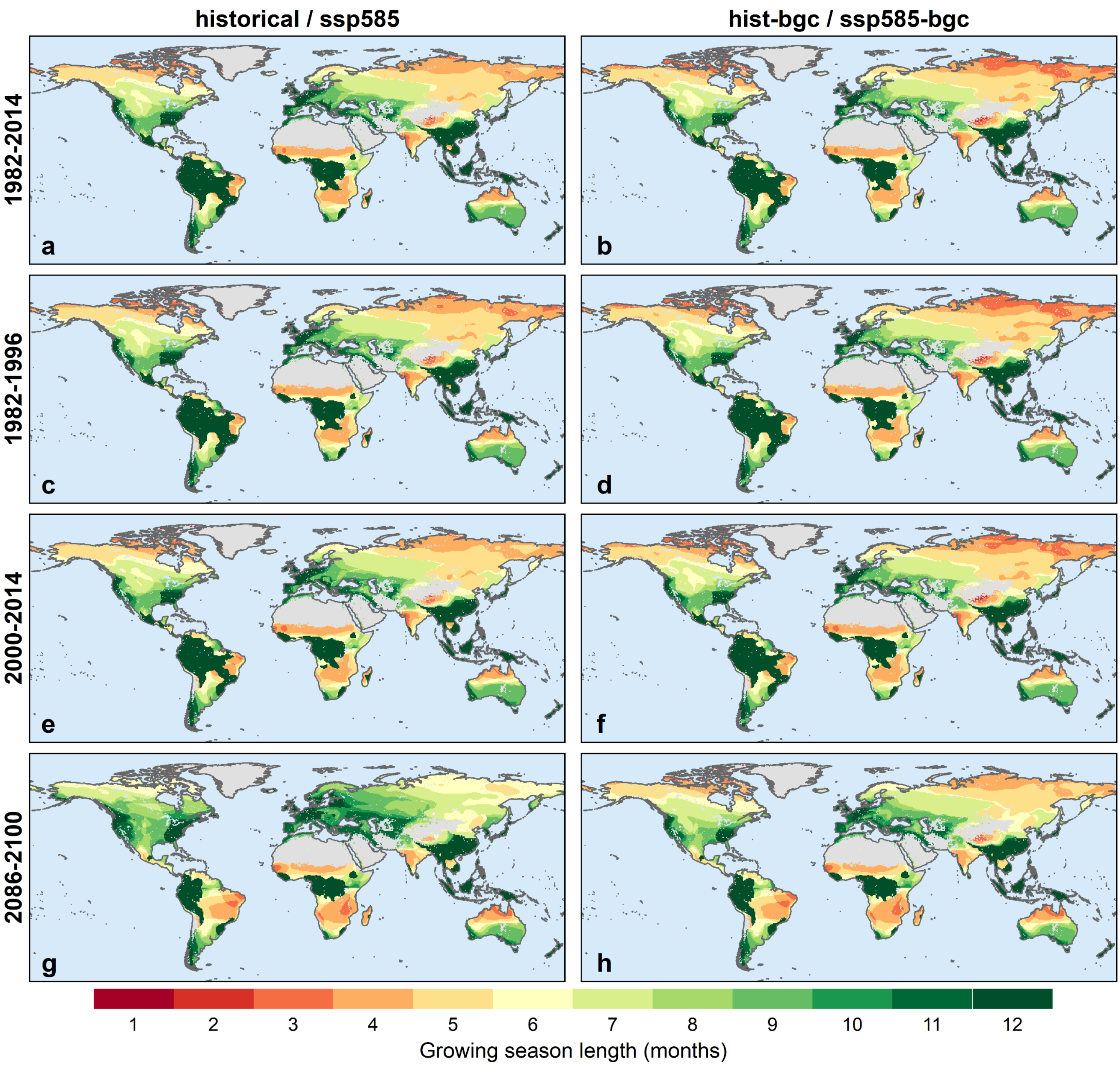

Supplementary Fig. 20 Vegetation phenology for different periods. (a and b) Spatial pattern of growing season length in months during the period 1982-2014 in the fully-couple experiment and in the biogeochemically-coupled experiment derived from $CMIP6_{SMA}$. (c-h) Same as (a and b), but for the periods 1982-1996, 2000-2014, and 2086-2100 under the SSP5-8.5 scenario, respectively.

.

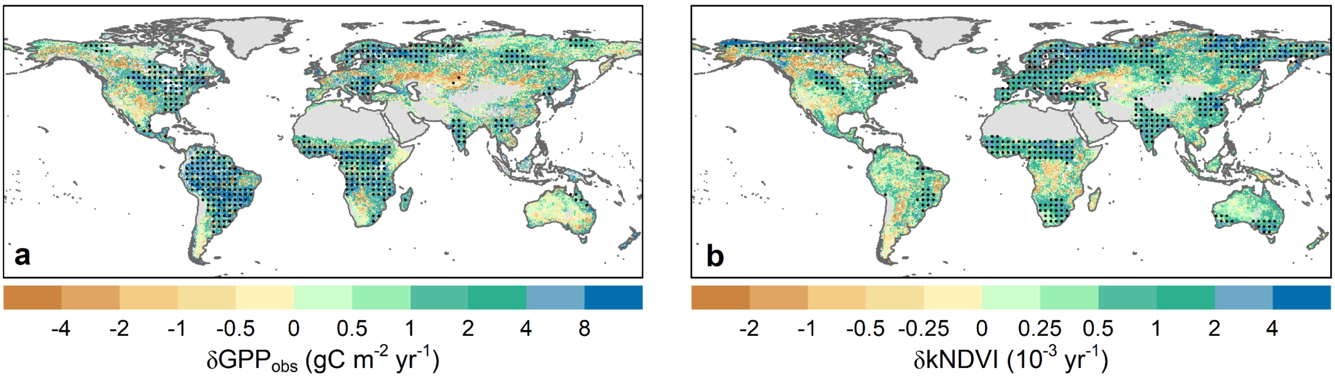

Supplementary Fig. 21 Observed trend in vegetation carbon uptake during the period 1982-2014. Spatial pattern of trend in growing-season (a) observed gross primary production (GPP_{obs}) derived from near-infrared reflectance of vegetation (NIRv) and (b) kernel normalized difference vegetation index (kNDVI) for 1982-2014. Regions labelled by black dots indicate trends that are statistically significant (Mann–Kendall test, $p<0.05$). Dots are spaced 3° in both latitude and longitude, and statistics were computed over 9°×9° spatial moving windows.

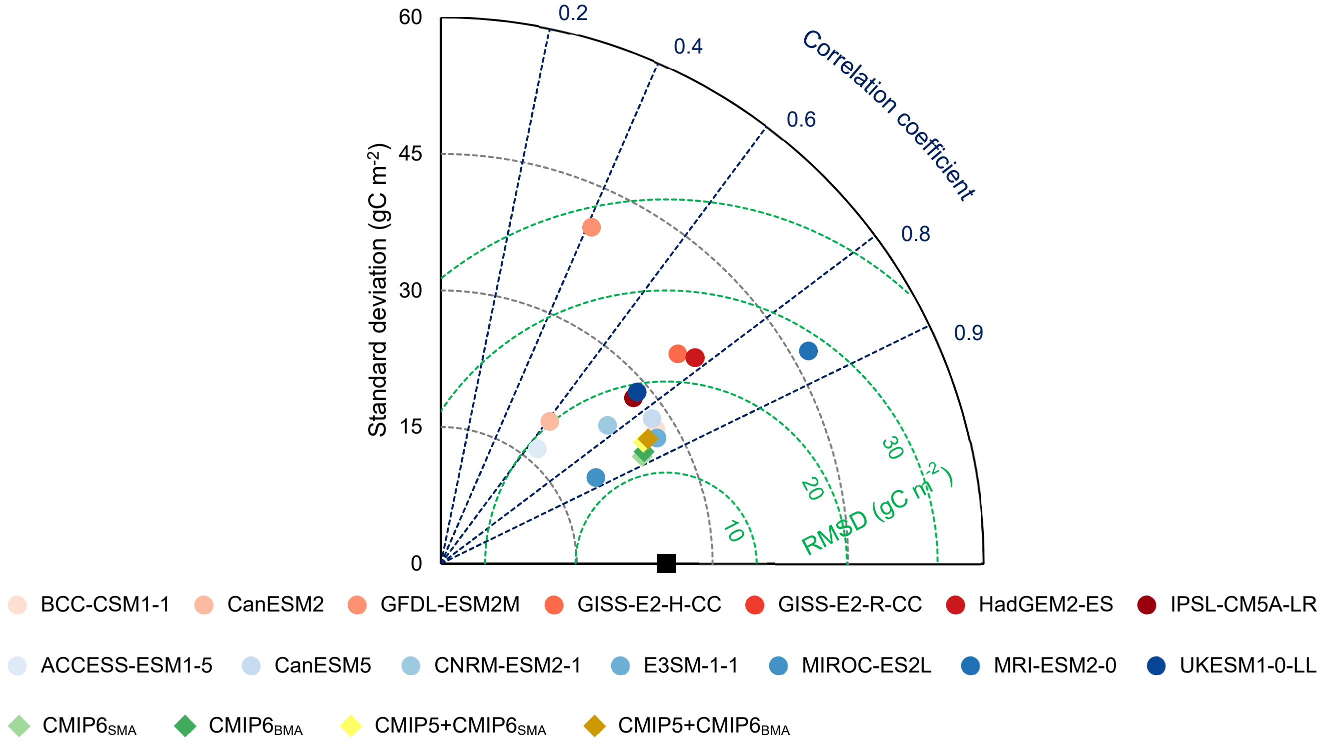

Supplementary Fig. 22 Taylor diagram compares the observed and model-simulated (under "historical" experiment) growing-season gross primary production (GPP) during the period 1982-2014 for global vegetated land. The standard deviation shows the interannual variability of the observed and the modeled GPP. The dash green lines show centered root mean square difference between model simulations and observation (i.e., GPP_{obs} derived from near-infrared reflectance of vegetation (NIRv)). Colored dots represent different ESMs used in this study, including BCC-CSM1-1, CanESM2, GFDL-ESM2M, GISS-E2-H-CC, GISS-E2-R-CC, HadGEM2-ES, IPSL-CM5A-LR, ACCESS-ESM1-5, CanESM5, CNRM-ESM2-1, E3SM-1-1, MIROC-ES2L, and UKESM1-0-LL (Table 1 and Supplementary Table 1), and their multiple-model ensemble means (i.e., $CMIP6_{SMA}$, $CMIP6_{BMA}$, $CMIP5+CMIP6_{SMA}$, and $CMIP5+CMIP6_{BMA}$). $CMIP6_{SMA}$ represents ensemble of seven CMIP6 ESMs integrated by simple model averaging, and $CMIP5+CMIP6_{BMA}$ represents ensemble of seven CMIP5 ESMs and seven CMIP6 ESMs integrated by Bayesian model averaging, and so on.

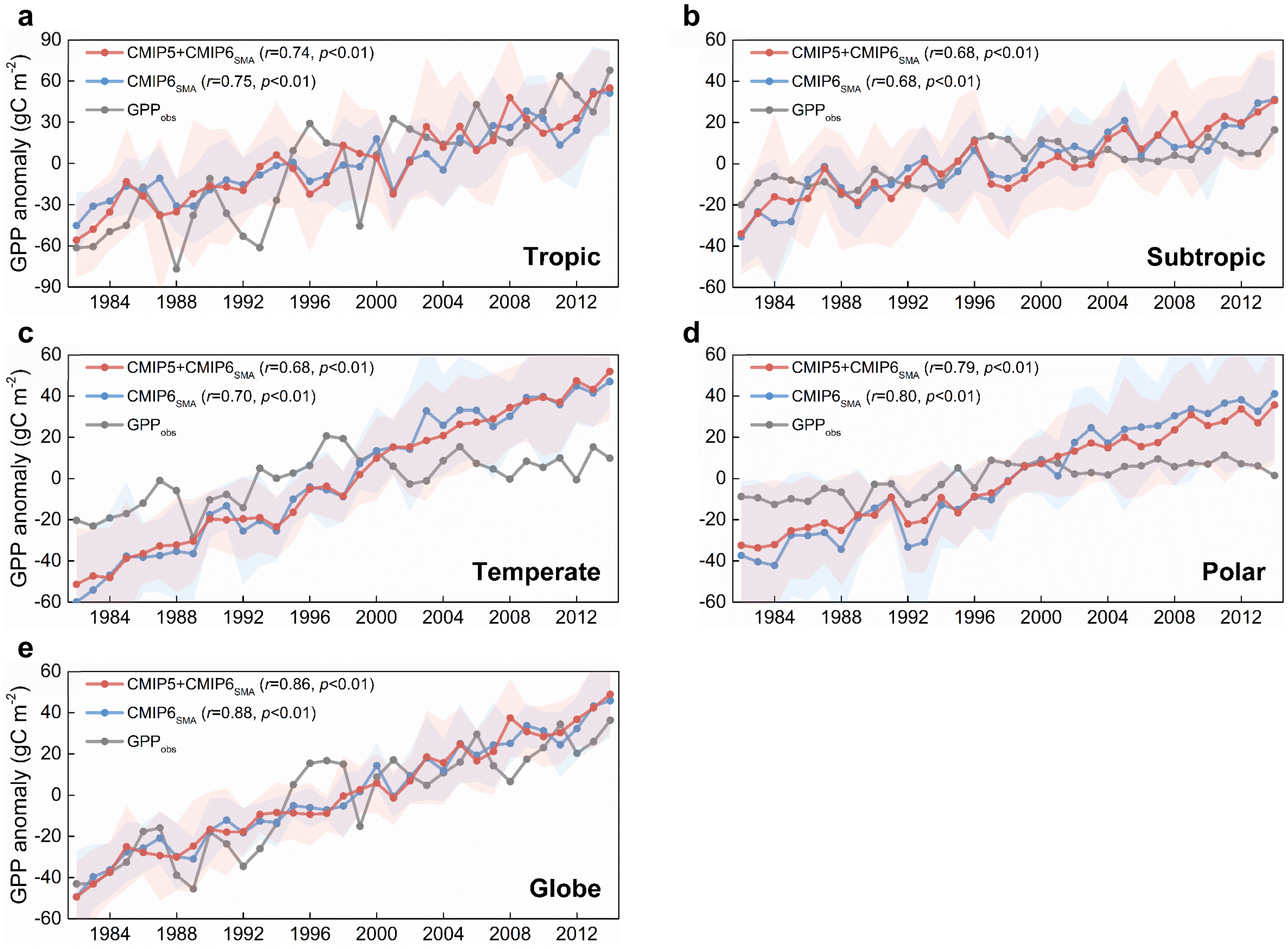

Supplementary Fig. 23 Interannual changes in anomalies of growing-season gross primary production (GPP) during the period 1982-2014. Blue and red lines represent time series of GPP simulated by ensemble mean of CMIP6 and CMIP5 ESMs (i.e., $CMIP6_{SMA}$ and CMIP5+$CMIP6_{SMA}$) in the fully-coupled experiment ("historical") and, grey lines represent those derived from the satellite observation (i.e., GPP_{obs} derived from near-infrared reflectance of vegetation (NIRv)) for the (a) tropical zone (23.5°S-23.5°N), (b) subtropical zone (23.5°S-35°S and 23.5°N-35°N), (c) temperate zone (35°S-66.5°S and 35°N-66.5°N), (d) polar zone (66.5°N-90°N), and (e) globe, respectively. Error bars represent the standard deviation of trends derived from model simulations. Source data are provided as a Source Data file.

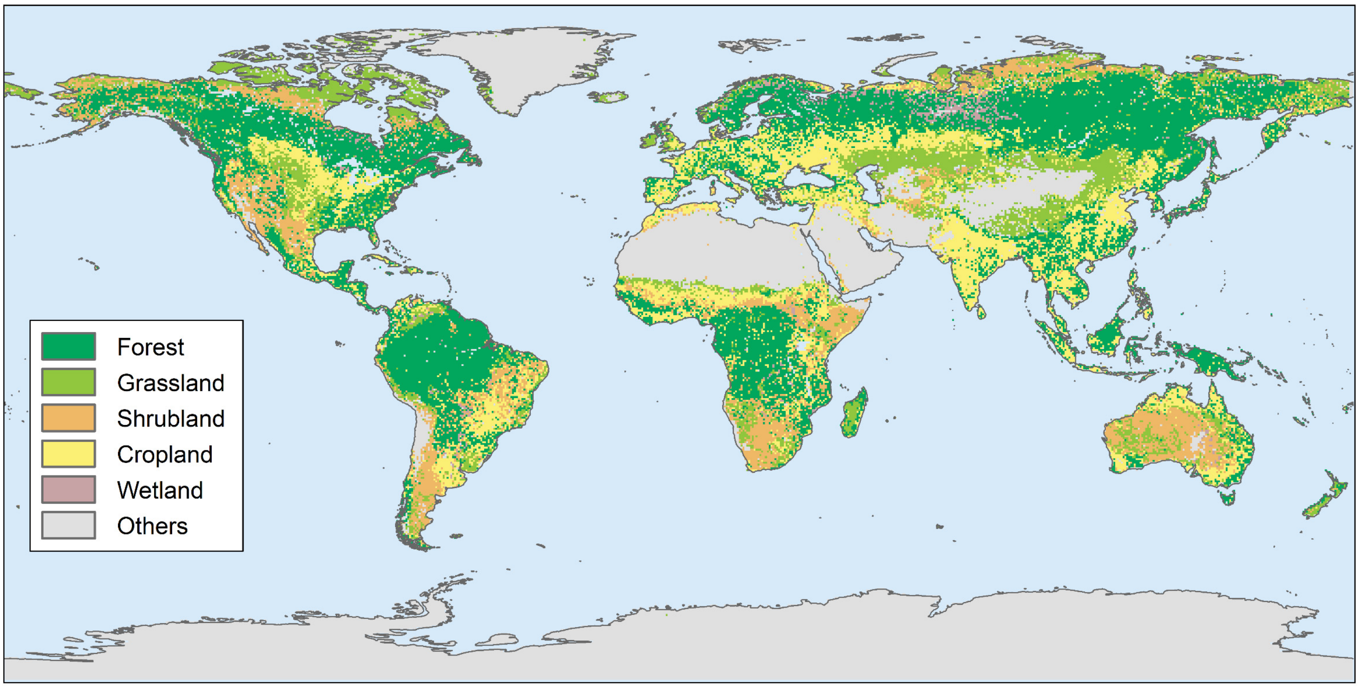

Supplementary Fig. 24 Land cover map at the 0.5°×0.5° spatial resolution. This map was derived from the ESA-CCI, comprising forest, grassland, shrubland, cropland, wetland, and other non-vegetated land (e.g., urban area, bare area, and permanent snow and ice). Areas with no vegetation cover are in grey. In this study, only vegetated land is considered for subsequent analysis.

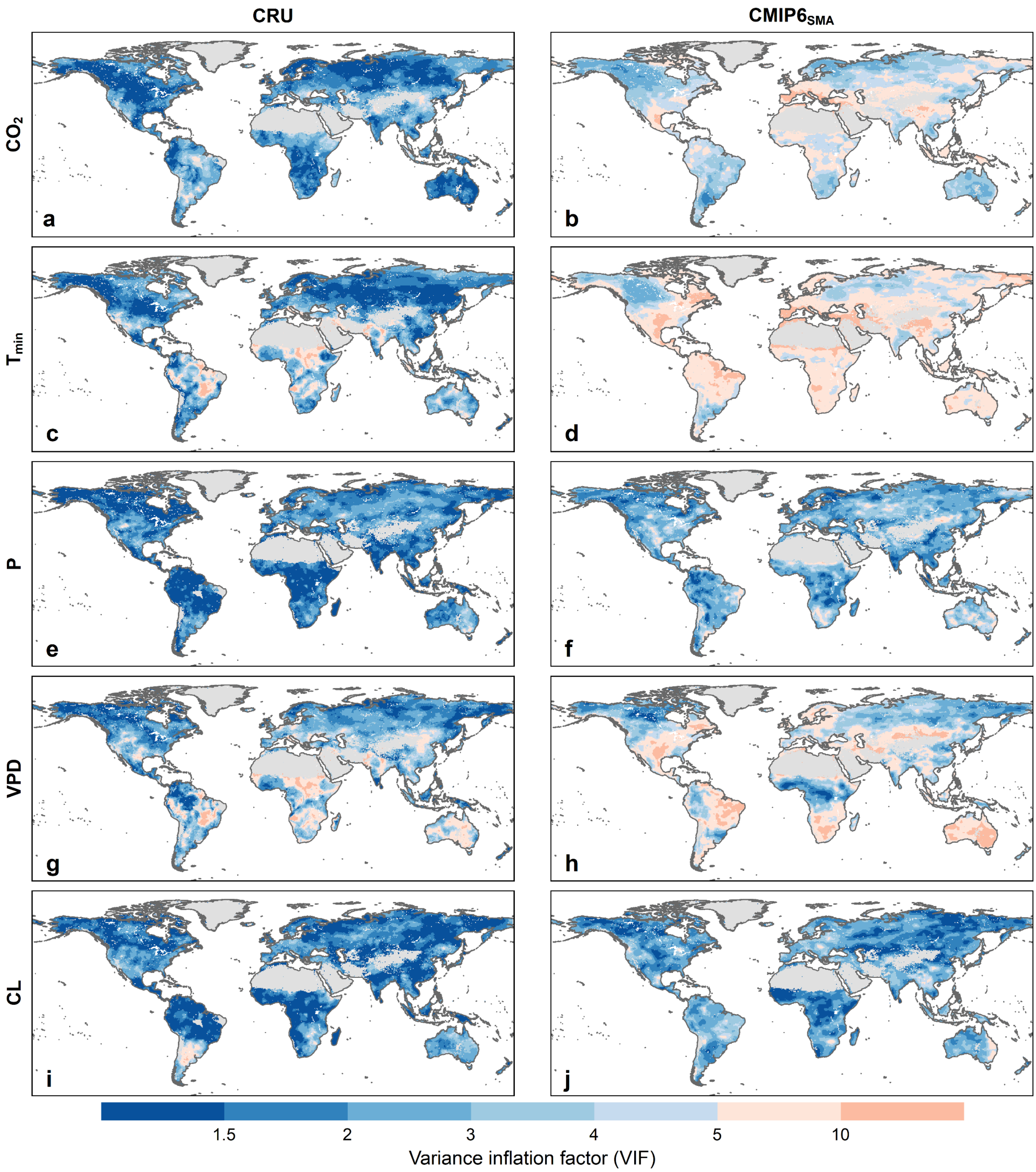

Supplementary Fig. 25 Multicollinearity test based on the variance inflation factor (VIF). Global VIF between (a and b) annual atmospheric CO_2 concentration, (c and d) minimum air temperature (T_{min}), (e and f) precipitation (P), (g and h) vapor pressure deficit (VPD), and (i and j) cloud cover (CL). T_{min}, P, and CL were obtained from (c, e, and i) CRU v4.05 dataset and (d, f, and j) ensemble mean of CMIP6 model outputs ($CMIP6_{SMA}$), respectively. Correspondingly, (g) CRU VPD was calculated by Eq. (2) and (h) CMIP6 VPD was calculated by Eq. (1). VIF provides a measure of multicollinearity among the independent variables, and a high VIF (>10 in general) indicates that the associated independent variable is highly collinear with the other variables.

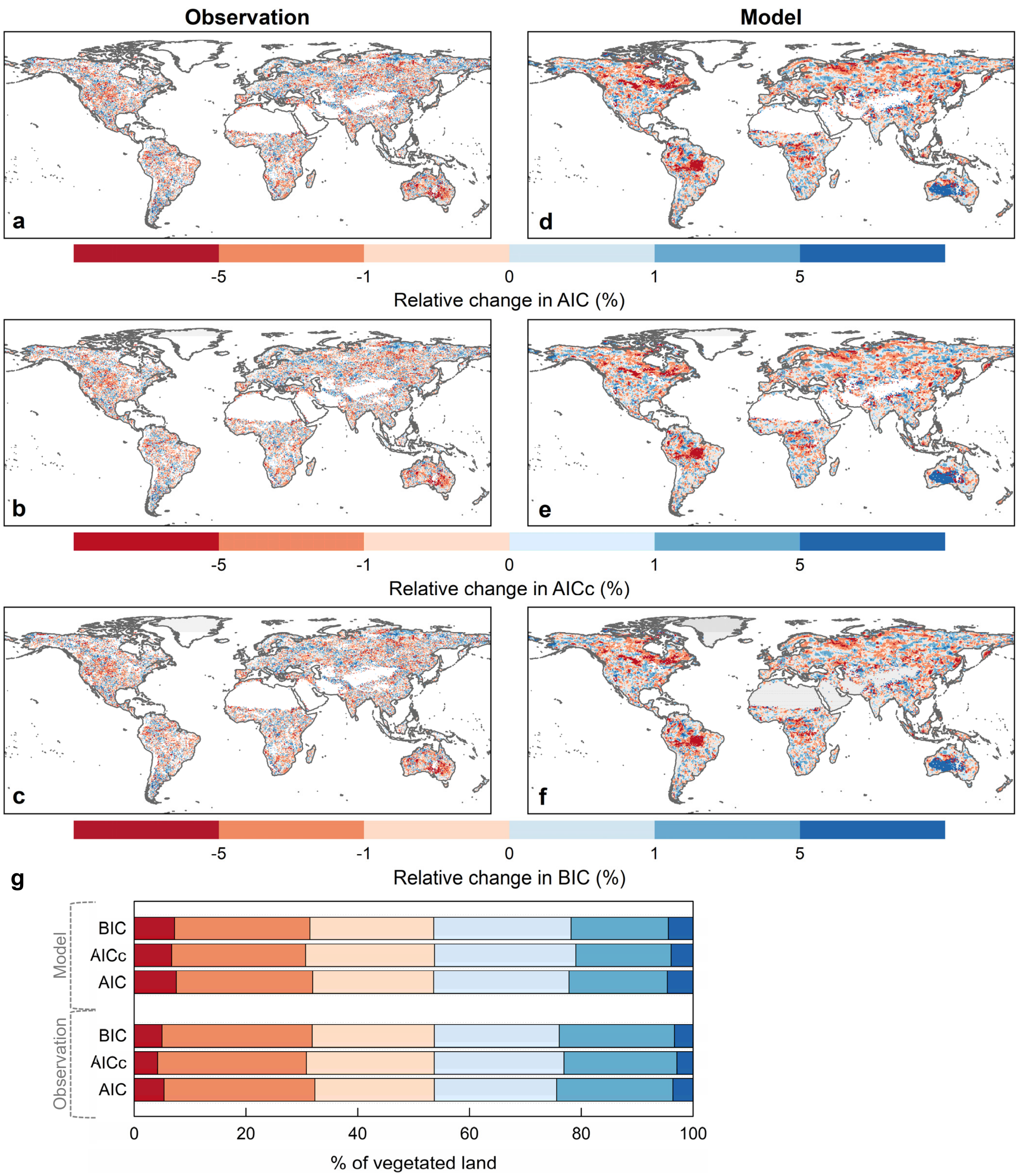

Supplementary Fig. 26 Performance evaluation and comparison of two different non-linear regression models. (a-c) Performance comparison of Eq. (4) and Eq. (19) with observation-based datasets as inputs for the period 1982-1996 on the basis of the Akaike Information Criterion (AIC), the corrected Akaike Information Criterion (AICc) and the Bayesian Information Criterion (BIC). Relative change in AIC was calculated by $\left(AIC_{Eq.(19)} - AIC_{Eq.(4)}\right)/AIC_{Eq.(4)}$. (d-f) Same as (a-c), but for Eq. (4) and Eq. (19) with multi-model simulations as inputs. (g) Fraction of vegetated land with different levels of relative change in AIC, AICc and BIC.

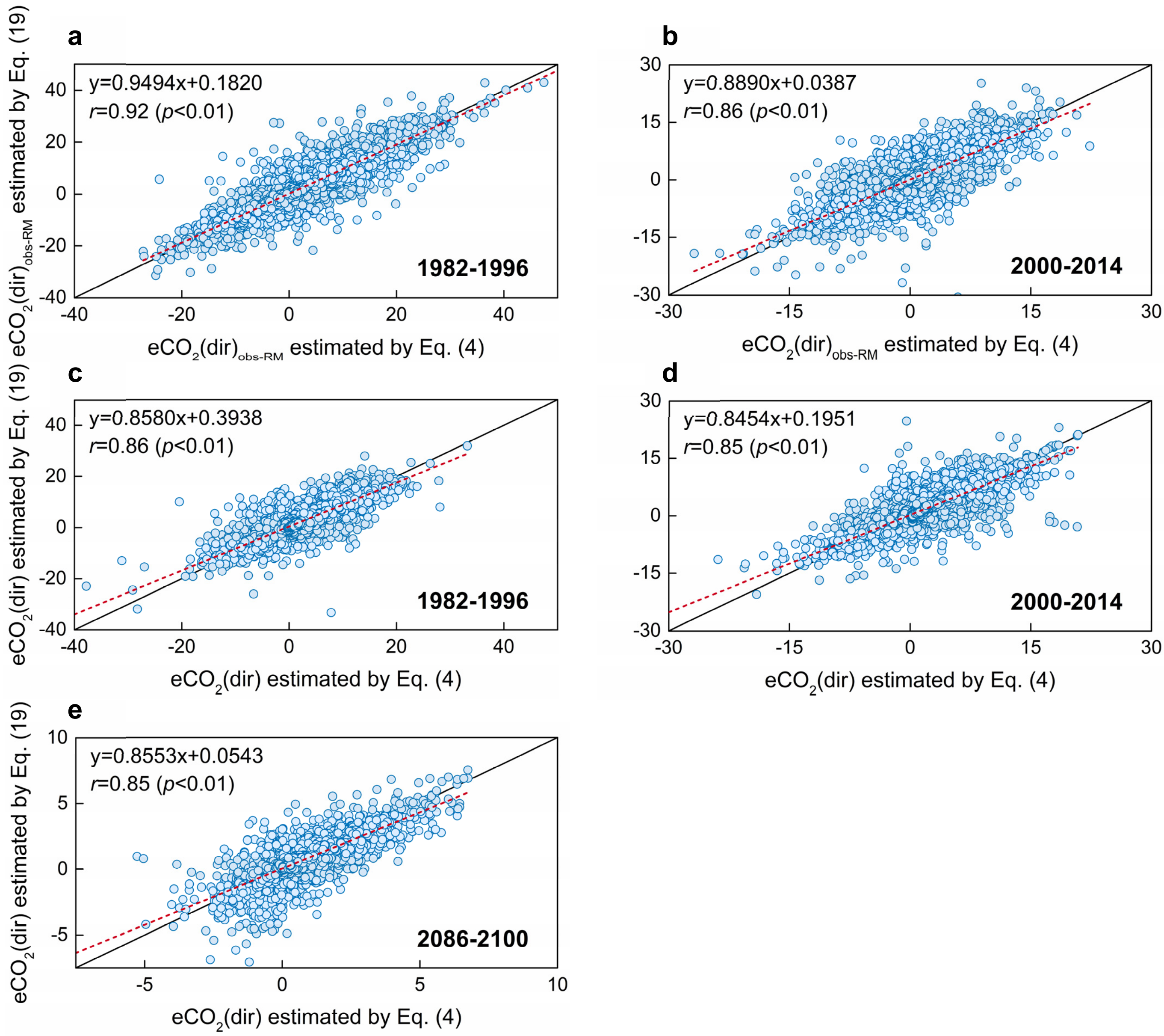

Supplementary Fig. 27 Comparison of estimates on direct CO_2 effect based on different methods. Comparisons of direct (physiological) effect of elevated atmospheric CO_2 concentration (eCO_2) on growing-season gross primary production (GPP) ($eCO_2(dir)_{obs\text{-}RM}$) during the periods (a) 1982-1996 and (b) 2000-2014 estimated by Eq. (19) against that estimated by Eq. (4) with observation-based datasets as inputs. Each symbol represents one vegetated grid-cell. Red dotted lines indicate the best-fit with equation provided on each subplot. (c-e) Same as (a and b), but for estimates based on multi-model ensemble simulations (i.e., $eCO_2(dir)$). Source data are provided as a Source Data file.

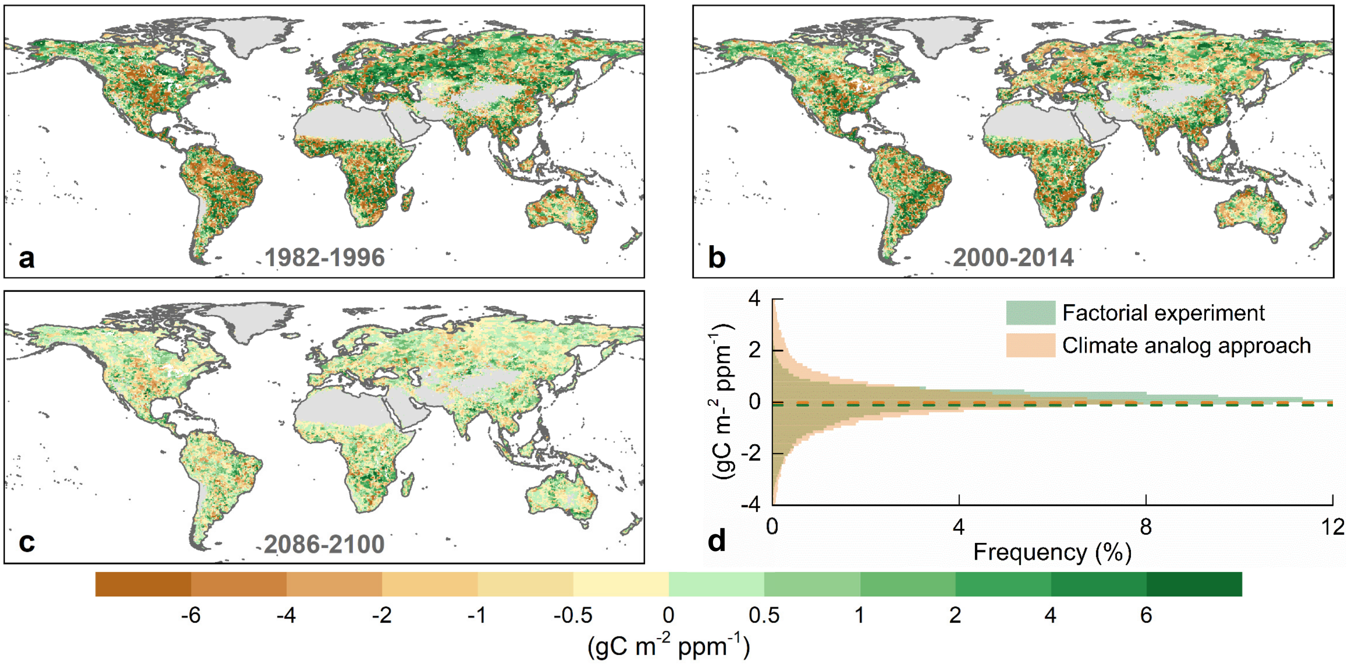

Supplementary Fig. 28 Indirect effect of elevated atmospheric CO_2 concentration (eCO_2) on vegetation carbon uptake calculated based on the combination of model outputs and the climate analog approach. (a-c) Spatial pattern of indirect effect of eCO_2 on growing-season gross primary production (GPP) via associated climate change (eCO_2(ind)) during the periods 1982-1996, 2000-2014, and 2086-2100 under the SSP5-8.5 scenario, based on model outputs from $CMIP6_{SMA}$ in combination with the temporal climate analog approach. (d) Frequency distribution of eCO_2(ind) over 2086-2100 projected directly by the CMIP6 factorial experiment (i.e., Eq. (3)), and that projected by CMIP6 model outputs in combination with the climate analog approach. Distribution averages are shown as dotted horizontal lines. Source data are provided as a Source Data file.

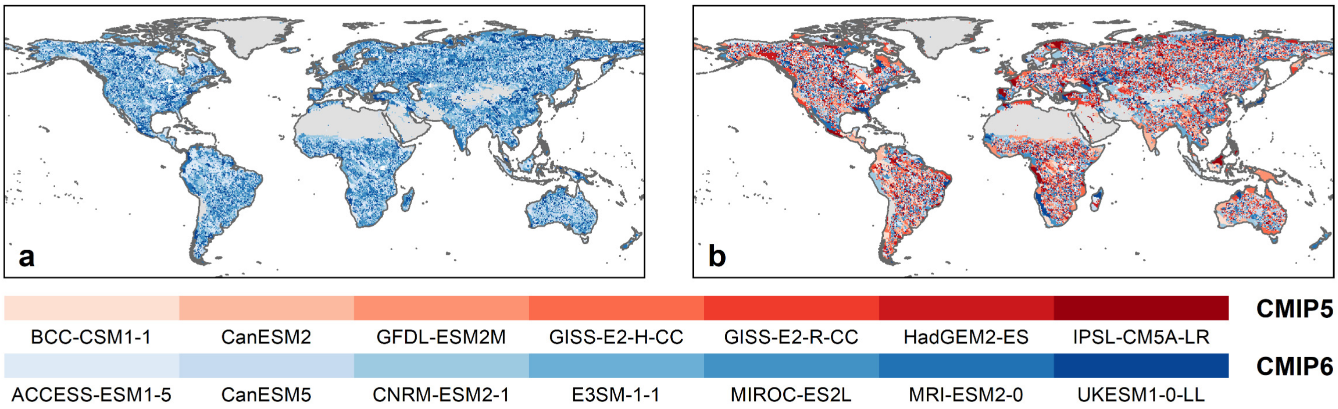

Supplementary Fig. 29 Assigned weights for models. Spatial pattern of the maximum optimal weights of the (a) seven CMIP6 ESMs and (b) total fourteen ESMs estimated by Bayesian model averaging (BMA).

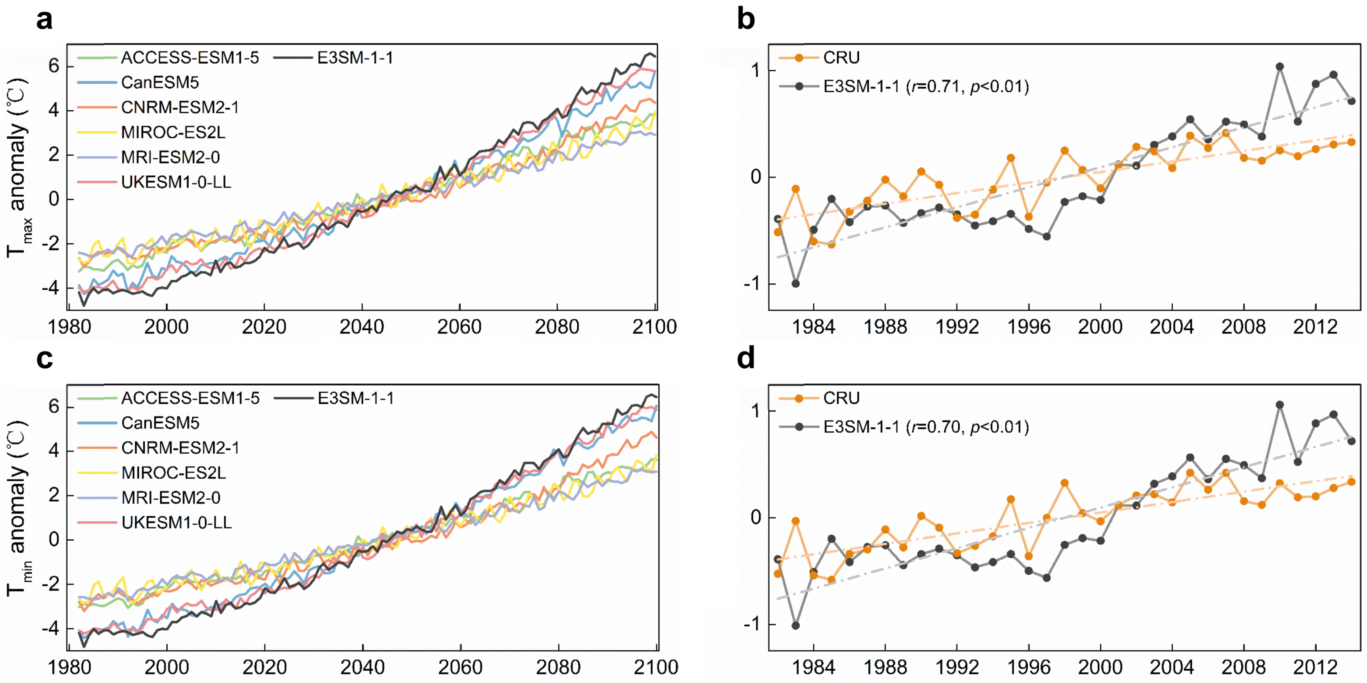

Supplementary Fig. 30 Interannual changes in anomalies of maximum air temperature (T_{max}) and minimum air temperature (T_{min}) at the global scale. (a) Time series of T_{max} anomalies simulated by seven CMIP6 ESMs under fully-coupled experiment ("historical" and "ssp585") during the period 1982-2100. (b) Time series of T_{max} anomalies simulated by E3SM-1-1 under fully-coupled experiment ("historical") and those derived from CRU climate dataset during the period 1982-2014. Dotted lines indicate linear regressions of time series of T_{max} anomalies. (c and d) Same as (a and b), but for T_{min}. T_{max} and T_{min} time series of E3SM-1-1 was estimated based on the regression relationship built by other six ESMs in combination with mean air temperature (T) provided by E3SM-1-1 (details in Supplementary Text 5). Source data are provided as a Source Data file.

Supplementary Table 1. Information of CMIP5 ESMs used in this study.

Model Name	Land Surface Component	Modelling Center
BCC-CSM1-1	BCC-AVIM1.0	Beijing Climate Center, China Meteorological Administration
CanESM2	CTEM	Canadian Centre for Climate Modelling and Analysis
GFDL-ESM2M	GFDL-LM3.0	NOAA Geophysical Fluid Dynamics Laboratory
GISS-E2-H-CC	GISS LSM	NASA Goddard Institute for Space Studies
GISS-E2-R-CC	GISS LSM	NASA Goddard Institute for Space Studies
HadGEM2-ES	TRIFFID	Met Office Hadley Centre
IPSL-CM5A-LR	ORCHIDEE	Institute Pierre Simon Laplace

Supplementary Table 2. Mean indirect (through associated climate change), direct physiological, and net effect of elevated atmospheric CO_2 concentration (eCO_2) on growing-season gross primary production (GPP) (i.e., eCO_2(ind), eCO_2(dir), and eCO_2(net), in unit of gC m^{-2} ppm^{-1}) during the period 1982-1996, 1982-2014, and 2086-2100 under the SSP5-8.5 scenario, for the globe, Northern Hemisphere, Southern Hemisphere, and Northern land (>50°), respectively, as estimated by CMIP6 model simulations. Values are expressed as ensemble mean ± standard error.

	1982-1996			1982-2014			2086-2100 under SSP5-8.5		
	eCO_2(ind)	eCO_2(dir)	eCO_2(net)	eCO_2(ind)	eCO_2(dir)	eCO_2(net)	eCO_2(ind)	eCO_2(dir)	eCO_2(net)
Globe	0.24±0.32	1.93±0.28	2.17±0.42	0.14±0.08	1.83±0.24	1.98±0.31	-0.12±0.09	0.65±0.12	0.53±0.11
Northern Hemisphere	0.43±0.25	1.85±0.39	2.27±0.41	0.35±0.11	1.81±0.30	2.16±0.40	-0.02±0.08	0.56±0.09	0.54±0.11
Southern Hemisphere	-0.19±0.78	2.13±0.20	1.94±0.89	-0.33±0.13	1.89±0.31	1.57±0.34	-0.34±0.19	0.84±0.20	0.50±0.18
Northern land (>50°N)	1.23±0.48	2.05±0.51	3.28±0.67	1.01±0.24	2.04±0.52	3.05±0.75	0.13±0.11	0.46±0.07	0.59±0.14

Supplementary Table 3. Global mean direct physiological effect of elevated atmospheric CO_2 concentration (eCO_2) on growing-season gross primary production (GPP) (i.e., eCO_2(dir), in unit of gC m^{-2} ppm^{-1}) during the periods 1982-1996, 2000-2014, 1982-2014, and 2086-2100, as estimated by 7-member model ensemble simulations in combination with Eq. (4) and Eq. (19), respectively. Values are expressed as ensemble mean ± standard error.

	1982-1996	2000-2014	1982-2014	2086-2100
Eq. (4)	1.93±0.28	1.49±0.12	1.83±0.24	0.65±0.12
Eq. (19)	2.05±0.29	1.47±0.14	1.87±0.27	0.61±0.15

Supplementary References

1. Green, J. et al. Large influence of soil moisture on long-term terrestrial carbon uptake. *Nature* **565**, 476-479 (2019).
2. Zhu, Z. et al. Attribution of seasonal leaf area index trends in the northern latitudes with "optimally" integrated ecosystem models. *Glob. Change Biol.* **23**, 4798-4813 (2017).
3. Duan, Q., Ajami, N., Gao, X. & Sorooshian, S. Multi-model ensemble hydrologic prediction using Bayesian model averaging. *Adv. Water Resour.* **30**, 1371-1386 (2007).
4. Sakia, R. The box-cox transformation technique: A review. *J. R. Stat. Soc. D* **41**, 169-178 (1992).
5. Taylor, K., Stouffer, R. & Meehl, G. An overview of CMIP5 and the experiment design. *Bull. Am. Meteorol. Soc.* **93**, 485-498 (2011).
6. Kim, M. et al. Performance evaluation of CMIP5 and CMIP6 models on heatwaves in Korea and associated teleconnection patterns. *J. Geophys. Res. Atmos.* **125**, e2020JD032583 (2020).
7. Tokarska, K. et al. Past warming trend constrains future warming in CMIP6 models. *Sci. Adv.* **6**, eaaz9549 (2020).
8. Yuan, S., Quiring, S. & Leasor, Z. Historical changes in surface soil moisture over the contiguous United States: An assessment of CMIP6. *Geophys. Res. Lett.* **47**, e2020GL089991 (2021).
9. Pinzon, J. & Tucker, C. A non-stationary 1981–2012 AVHRR NDVI3g time series. *Remote Sens.* **6(8)**, 6929-6960 (2014).
10. Camps-Valls, G. et al. A unified vegetation index for quantifying the terrestrial biosphere. *Sci. Adv.* **7**, eabc7447 (2021).
11. Forzieri, G., Dakos, V., McDowell, N., Ramdane, A. & Cescatti, A. Emerging signals of declining forest resilience under climate change. *Nature* **608**, 534-539 (2022).
12. Jones, C. et al. C4MIP – The Coupled Climate–Carbon Cycle Model Intercomparison Project: experimental protocol for CMIP6. *Geosci. Model Dev.* **9**, 2853–2880 (2016).
13. Allen, R. G., Pereira, L. S., Raes, D. & Smith, M. Crop Evapotranspiration-Guidelines for Computing Crop Water Requirements. FAO Irrigation and Drainage Paper No. 56 (1998).
14. Trenberth, K. et al. Global warming and changes in drought. *Nat. Clim. Change* **4**, 17-22 (2014).
15. Huang, J., Yu, H., Guan, X., Wang, G. & Guo, R. Accelerated dryland expansion under climate change. *Nat. Clim. Change* **6**, 166-171 (2016).
16. Wang, W., Li, C., Xing, W. & Fu, J. Projecting the potential evapotranspiration by coupling different formulations and input data reliabilities: The possible uncertainty source for climate change impacts on hydrological regime. *J. Hydrol.* **555**, 298-313 (2017).
17. Gentine, P. et al. Coupling between the terrestrial carbon and water cycles—a review. *Environ. Res. Lett.* **14**, 083003 (2019).
18. Martens, B. et al. GLEAM v3: satellite-based land evaporation and root-zone soil moisture. *Geosci. Model Dev.* **10**, 1903-1925 (2017).
19. Trugman, A., Medvigy, D., Mankin, J. & Anderegg, W. Soil moisture stress as a major driver of carbon cycle uncertainty. *Geophys. Res. Lett.* **45**, 6495–6503 (2018).
20. Li, W. et al. Widespread increasing vegetation sensitivity to soil moisture. *Nat. Commun.* **13**, 3959 (2022).
